# Latent Profiles of Childhood Adversity, Adolescent Mental Health, and Neural Network Connectivity

**DOI:** 10.1001/jamanetworkopen.2024.30711

**Published:** 2024-08-28

**Authors:** Felicia A. Hardi, Adriene M. Beltz, Vonnie McLoyd, Jeanne Brooks-Gunn, Edward Huntley, Colter Mitchell, Luke W. Hyde, Christopher S. Monk

**Affiliations:** 1 Department of Psychology, University of Michigan, Ann Arbor; 2 Yale University, New Haven, Connecticut; 3 Survey Research Center of the Institute for Social Research, University of Michigan, Ann Arbor; 4 Population Studies Center of the Institute for Social Research, University of Michigan, Ann Arbor; 5 Teachers College, Columbia University, New York, New York; 6 College of Physicians and Surgeons, Columbia University, New York, New York; 7 Neuroscience Graduate Program, University of Michigan, Ann Arbor; 8 Department of Psychiatry, University of Michigan, Ann Arbor

## Abstract

**Importance:**

Adverse childhood experiences are pervasive and heterogeneous, with potential lifelong consequences for psychiatric morbidity and brain health. Existing research does not capture the complex interplay of multiple adversities, resulting in a lack of precision in understanding their associations with neural function and mental health.

**Objectives:**

To identify distinct childhood adversity profiles and examine their associations with adolescent mental health and brain connectivity.

**Design, Setting, and Participants:**

This population-based birth cohort used data for children who were born in 20 large US cities between 1998 and 2000 and participated in the Future Families and Child Well-Being Study. Families were interviewed when children were born and at ages 1, 3, 5, 9, and 15 years. At age 15 years, neuroimaging data were collected from a subset of these youths. Data were collected from February 1998 to April 2017. Analyses were conducted from March to December 2023.

**Exposures:**

Latent profiles of childhood adversity, defined by family and neighborhood risks across ages 0 to 9 years.

**Main Outcomes and Measures:**

Internalizing and externalizing symptoms at age 15 years using parent- and youth-reported measures. Profile-specific functional magnetic resonance imaging connectivity across the default mode network (DMN), salience network (SN), and frontoparietal network (FPN).

**Results:**

Data from 4210 individuals (2211 [52.5%] male; 1959 [46.5%] Black, 1169 [27.7%] Hispanic, and 786 [18.7%] White) revealed 4 childhood adversity profiles: low-adversity (1230 individuals [29.2%]), medium-adversity (1973 [46.9%]), high-adversity (457 [10.9%]), and high maternal depression (MD; 550 [13.1%]). High-adversity, followed by MD, profiles had the highest symptoms. Notably, internalizing symptoms did not differ between these 2 profiles (mean difference, 0.11; 95% CI, −0.03 to 0.26), despite the MD profile showing adversity levels most similar to the medium-adversity profile. In the neuroimaging subsample of 167 individuals (91 [54.5%] female; 128 [76.6%] Black, 11 [6.6%] Hispanic, and 20 [12.0%] White; mean [SD] age, 15.9 [0.5] years), high-adversity and MD profiles had the highest DMN density relative to other profiles (*F*_(3,163)_ = 11.14; *P* < .001). The high-adversity profile had lower SN density relative to the low-adversity profile (mean difference, −0.02; 95% CI, −0.04 to −0.003) and the highest FPN density among all profiles (*F*_(3,163)_ = 18.96; *P* < .001). These differences were specific to brain connectivity during an emotion task, but not at rest.

**Conclusions and Relevance:**

In this cohort study, children who experienced multiple adversities, or only elevated MD, had worse mental health and different neural connectivity in adolescence. Interventions targeting multiple risk factors, with a focus on maternal mental health, could produce the greatest benefits.

## Key Points

**Question:**

What patterns of adolescent mental health and brain function emerge from profiles of childhood adversity?

**Findings:**

In this cohort study of 4210 youths examined over 15 years, data-driven clustering and person-specific network neuroscience approaches were combined to assess the mental health and brain function associated with 4 identified childhood adversity profiles. These profiles had different symptomatic presentations and network connectivity, with youths exposed to high multidomain adversity and maternal depression exhibiting heightened symptoms.

**Meaning:**

The findings of this study suggest that more consideration for individual differences in adverse experiences across multiple domains, particularly maternal depression, is needed for targeted interventions addressing adolescent mental health.

## Teaser

This cohort study evaluates whether different childhood adversity profiles are associated with mental health in adolescence and measures of brain connectivity among US youths.

## Introduction

Adverse childhood experiences are prevalent risk factors for health across the lifespan and are associated with nearly 30% of all psychiatric disorders.^1,2,3^ Childhood adversity has been linked with differences in brain function during emotion processing in human and animal research,^4,5,6,7,8,9^ thus providing insight into how adversity could disrupt critical domains of development that contribute to psychopathology later in life.

Adverse experiences, ranging from maltreatment and family violence to household instability and community violence, often co-occur and interact.^2,3,10,11,12^ Nevertheless, exposure to one adversity does not necessarily indicate the presence of another,^3,11^ underscoring the broad heterogeneity of the profiles of exposure to various adverse environments. However, research examining the neural correlates of adversity has typically focused on singular exposures or cumulative indices of exposures^3,10,13^ without considering how different combinations of risks may uniquely influence mental health. Cumulative models assume that each adverse experience operates in a similar manner and holds equal importance for each individual,^14,15,16,17^ which undercuts the identification of precise adversity-linked neural correlates that can ultimately improve prevention and interventions.

Thus, more work is needed to parse heterogeneity within individuals’ multifaceted adverse environments. To improve precision in clinical interventions, a growing body of work has sought to identify latent subpopulations that share similar characteristics.^18,19,20^ While these person-oriented approaches have largely been applied to classify subgroups of individuals with complex health outcomes,^21,22,23,24,25^ they have not focused on explaining variations in adolescent mental health and brain function simultaneously, specifically in population-based samples with rich contextual information about adverse experiences across multiple developmental years. Additionally, person-centered clustering methods, which identify data-driven hidden classes or subgroups of individuals, can be combined with person-specific network neuroscience methods that allow for the estimation of neural patterns across the group, subgroup, and individual levels,^26,27^ increasing the ability to reveal commonalities and differences within the population and capturing more granularity in modeling individual processes.

Work in clinical neuroscience postulates that disrupted communication within brain networks such as the default mode network (DMN),^28^ salience network (SN),^29^ and frontoparietal network (FPN)^30^ underlies vulnerabilities to psychiatric disorders.^31^ A variety of adversities has been found to be differentially associated with these neural networks during rest^8,32^; however, the extent to which these networks differ among distinct broad-based adverse environments during active emotion processing is unknown. Neuroimaging data collected during behavioral tasks may characterize neural patterns that are more representative of processes implicated in psychopathology, thus improving the prediction of clinical traits.^33,34^

This study aimed to characterize heterogeneity in mental health and network function during emotion processing among subgroups of youths with different profiles of childhood adversity. Utilizing longitudinal data from a population-based birth cohort sample that includes a substantial proportion of individuals with marginalized and underrepresented identities,^35^ individuals were clustered based on childhood adversity exposure (0-9 years) across multiple contexts. The resulting profiles were examined in association with adolescent mental health (age 15 years). This clustering method was then combined with a person-specific connectivity approach that estimated profile-specific emotion-linked network patterns within a neuroimaging subsample (age 15 years).

## Methods

### Setting and Participants

Participants were from the Future Families and Child Well-Being study, a birth cohort population-based sample of children born in 20 large US cities (population >200 000) between 1998 and 2000, with an oversampling (3:1) of nonmarital births.^36^ Data from ages 1, 3, 5, 9, and 15 years were included. Participants who did not reside with the mother at least half of the time at any point (n = 290) and those with adversity data at fewer than 2 time points were excluded (n = 398). There were no differences between sample demographics (eTable 1 in Supplement 1). Response rates (as a percentage of the baseline sample) averaged 84% over 5 waves (year 1, 91%; year 3, 89%; year 5, 88%; year 9, 78%; year 15, 74%). Families not participating in one wave could participate in subsequent waves. At year 15, a cohort of families participated in the Study of Adolescent Neural Development at the University of Michigan, Ann Arbor, where neuroimaging data were collected. After exclusions for scanning ineligibility and preprocessing quality control (n = 63) (eFigure 1 in Supplement 1), the final neuroimaging sample included 167 individuals (eTable 2 in Supplement 1). The University of Michigan institutional review board approved the study, and caregivers and participants provided written informed consent (oral assent as minors) at all time points. This report followed the Strengthening the Reporting of Observational Studies in Epidemiology (STROBE) guidelines. Analyses ran from March to December 2023.

### Measures

#### Childhood Adversity

Ten indicators were selected to represent adverse childhood experiences within and outside of the home that contribute to youth mental health problems (eMethods in Supplement 1). These indicators were childhood maltreatment (emotional abuse, physical abuse, neglect; by Parent-Child Conflict Tactics Scale),^37^ intimate partner violence (by Relationship Quality Questionnaire),^38^ maternal depression (by Composite International Diagnostic Interview–Short Form),^39^ parental stress (by Parent Stress Inventory),^40^ residential moves (by frequency of moves between waves), neighborhood violence (by neighborhood violence questions),^41^ and lack of protective influences (community cohesion measured by Social Cohesion and Trust Scale and social control by Informal Social Control Scale).^42,43^

#### Functional Magnetic Resonance Imaging Data

Neuroimaging data were acquired using a 3T GE Discovery MR750 scanner with an 8-channel head coil. Two types of functional magnetic resonance imaging (fMRI) data were collected: while participants completed an in-scanner emotion task (task-based data), in which they were asked to identify the gender of the actor who was displaying affective facial expressions (fear, happy, sad, neutral, angry), and while participants were passively looking at a fixation cross (resting-state data) (eMethods in Supplement 1). Consistent with a previous investigation,^9^ task-based functional data were extracted across the entire task (including all emotion conditions and crosshair presentations), and standard fMRI preprocessing pipeline^44^ was applied using FSL version 6.0 (eMethods in Supplement 1). Preprocessed time series data were extracted from 9 bilateral regions of interest (ROIs) representing the DMN, SN, and FPN. Node coordinates were established using NeuroSynth^8,9,45^ (eTable 3 in Supplement 1).

#### Youth Mental Health Outcomes

Internalizing and externalizing problems were measured using second-order multi-informant latent factors, based on both parent and youth reports at age 15 years (eMethods in Supplement 1). Confirmatory factor analyses estimated the internalizing symptoms factor from 3 scales: parent-reported internalizing scale of the Child Behavioral Checklist (CBCL) 6-18,^46^ youth-reported items from the Brief Symptom Inventory 18,^47^ and youth-reported items from the Center for Epidemiologic Studies Depression Scale^48^ (eFigure 2 in Supplement 1). The externalizing behaviors factor was comprised of 3 scales: parent-reported externalizing scale of the CBCL,^46^ youth-reported items from the Delinquency scale,^49^ and youth-reported substance use (eFigure 3 in Supplement 1).

### Statistical Analysis

#### Latent Profile Analysis

Latent profile analysis (LPA) was performed on the full sample of 4210 participants using Mplus version 8.8 (Muthén and Muthén)^50^ to identify profiles of childhood adversity. LPA is a data-driven latent variable modeling approach that identifies hidden or unobserved subpopulations using a set of selected indicators (eg, multiple types of childhood adversity). In this study, profiles were identified using within-person mean exposure to various adversities from birth to age 9 years. Multiple model parameters (Akaike information criterion, bayesian information criterion, adjusted bayesian information criterion, Lo-Mendell-Rubin test) and classification characteristics (entropy, average posterior probabilities) were compared to determine the most parsimonious best-fitting model^51,52,53,54,55^ (eMethods and eTables 5-7 in Supplement 1). Missing data were addressed using maximum likelihood estimation with robust standard errors. To confirm the stability of the results, LPA on the selected number of classes was repeated for a total of 20 supplementary analyses, leaving out one site at a time (eMethods and eTable 8 in Supplement 1).

#### Estimation of Profile-Specific Functional Network Connectivity

In the neuroimaging subsample, person-specific functional connectivity was estimated for each latent profile using confirmatory subgrouping group iterative multiple model estimation (GIMME) using the gimme package in R version 4.2.1 (R Project for Statistical Computing). GIMME iteratively estimates connections among preselected ROIs using a unified structural equation model framework that includes estimation of group-level (present for at least 75% of all individuals), subgroup-level (present for at least 50% of individuals in each profile subgroup), and individual-level (present for each individual) connections^26,27^ (eMethods in Supplement 1).

Two types of connectivity metrics were computed: overall density (ie, network connectivity across all nodes) and density specific to each network (ie, DMN, SN, FPN). Network density was represented as a proportion of corresponding connections (eg, number of connections involving all DMN ROIs) from the overall network connections. Procedures were first applied to task-based neuroimaging data. Then, to determine that the resulting functional connectivity networks were unique to emotion-related processes, GIMME analyses were repeated using resting-state fMRI data and compared with the task-based results (eMethods in Supplement 1).

#### Analyses Examining Symptom and Network Variations by Adversity Profile

A 1-way analysis of variance was used to test differences among adversity profiles in both internalizing and externalizing symptoms and connectivity metrics (overall, DMN, SN, FPN density). Hypothesis tests were 2-sided with α = .05. Pairwise comparisons were conducted with adjustment for multiple comparisons using the Tukey-Kramer test. Sensitivity analyses with covariates (racial and ethnic identity, parental marital status, and household income) were conducted to adjust models for important sociodemographic differences. Race and ethnicity was youth-reported at age 15 years. For those youths who did not participate in wave 15, the mother’s baseline self-reported race and ethnicity was used to describe the sample (eMethods in Supplement 1). Racial and ethnic groups included Black non-Hispanic, Hispanic, multiracial non-Hispanic, White non-Hispanic, and other, the definition of which is not publicly available. Additional covariates were also included in the neuroimaging subsample analysis: youth age during the neuroimaging scan and in-scanner motion. Analyses examining profile differences across mental health outcomes and brain networks were then repeated separately for male and female participants (sex was parent-reported at birth) to consider sex as a biological variable (eMethods in Supplement 1).

## Results

### Adversity Latent Profiles

Of the 4210 participants included in the identification of childhood adversity latent profiles, 2211 (52.5%) were male, and 1959 (46.5%) were Black non-Hispanic, 1169 (27.7%) Hispanic, 156 (3.7%) multiracial non-Hispanic, 786 (18.7%) White non-Hispanic, and 136 (3.2%) other race and ethnicity. Of the 167 participants in the neuroimaging subsample (mean [SD] age, 15.9 [0.5] years), 91 (54.5%) were female, and 128 (76.6%) were Black non-Hispanic, 11 (6.6%) Hispanic, 4 (2.4%) multiracial non-Hispanic, 20 (12.0%) White non-Hispanic, and 4 (2.4%) other race and ethnicity.

**Figure 1.**
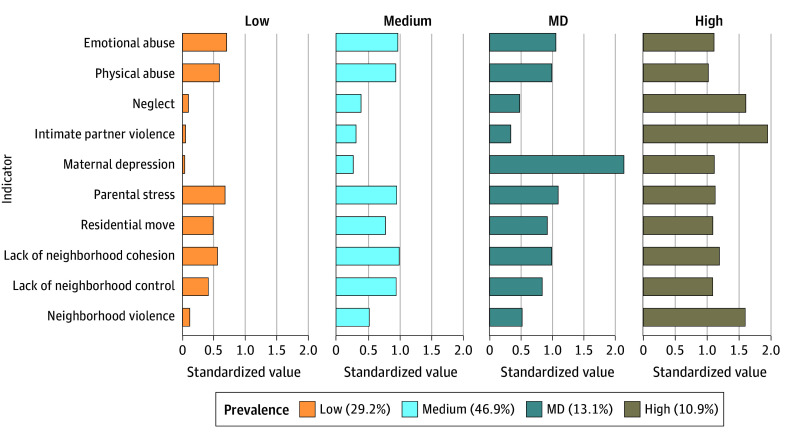
Standardized Values of Each Adversity Indicator in the 4-Class Model of Childhood Adversity Profiles Of 4210 individuals in the included sample, 1230 (29.2%) experienced the lowest rate of adversity, 1973 (46.9%) were exposed to medium-level adversity risk; and 457 (10.9%) experienced the highest level of risk across the 10 adversity types. A total of 550 individuals (13.1%) had similar levels of exposures with the medium-risk profile but with markedly elevated levels of maternal depression (MD) compared with all other profiles.

Zero-order correlations between adversity measures are in eTable 4 in Supplement 1 (range, *r* = 0.05 to *r* = 0.64). A 4-class model was the final selected model. Descriptive data appear in the Table and eTable 9 in Supplement 1; prevalence and adversity levels appear in Figure 1 as well as eTables 10 and 11 and eFigure 4 in Supplement 1). Profile 1 (1230 participants [29.2%]) had the lowest adversity scores across all indicators. Profiles 2 (1973 participants [46.9%]) and 3 (550 participants [13.1%]) showed moderate levels of adversity; however, profile 3 had a notably higher maternal depression (MD) rate. Profiles 2 and 3 did not differ in levels of physical abuse, neglect, intimate partner violence, lack of protective factors, and neighborhood violence (eTable 12 in Supplement 1). Differences between other indicators (emotional abuse, parental stress, residential move, lack of social control) were statistically significant but small in magnitude relative to MD. Profile 4 (457 participants [10.9%]) ranked highest in all adversities except for MD. To reflect these patterns, Profile 1 is termed low-adversity; profile 2, medium-adversity; profile 3, MD; and profile 4, high-adversity.

**Table.** Sociodemographic Characteristics of 4210 Participants, by Adversity Profile

Characteristic	Participants, No. (%)				Statistical test	*P* value
	Low-adversity (n = 1230)	Medium-adversity (n = 1973)	Maternal depression (n = 550)	High-adversity (n = 457)		
Race and ethnicity						
Black, non-Hispanic	434 (35)	1022 (52)	280 (51)	223 (49)	χ^2^_12_ = 239.4	<.001
Hispanic	337 (27)	572 (29)	104 (19)	156 (34)		
Multiracial, non-Hispanic	45 (4)	68 (3)	24 (4)	19 (4)		
White, non-Hispanic	368 (30)	252 (13)	131 (24)	35 (8)		
Other^a^	43 (3)	58 (3)	11 (2)	24 (5)		
Sex at birth						
Female	581 (47)	948 (48)	254 (46)	216 (47)	χ^2^_3_ = 0.66	.88
Male	649 (53)	1025 (52)	296 (54)	241 (53)		
Parental marital status						
Married	468 (38)	404 (20)	116 (21)	80 (18)	χ^2^_3_ = 149.64	<.001
Unmarried	762 (62)	1569 (80)	434 (79)	377 (82)		
Poverty ratio, mean (SD)^b^	3.25 (3.09)	1.98 (2.08)	2.01 (2.13)	1.52 (1.69)	*F*_(3,4206)_ = 95.31	<.001

^a^ Information about the other race and ethnicity subcategory is not publicly available.

^b^ Poverty ratio represents a ratio of total household income to the official poverty threshold at baseline (child birth).

### Profile Comparison in Youth Internalizing and Externalizing Symptoms

**Figure 2.**
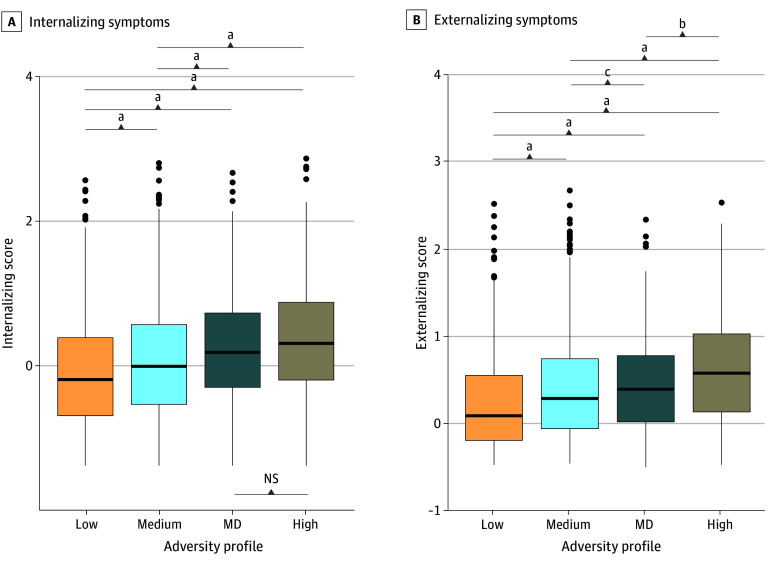
Boxplots Comparing Levels of Internalizing and Externalizing Symptoms The maternal depression (MD) and high-adversity profiles did not differ for internalizing symptoms but differed among all profiles for externalizing symptoms. Center line of box indicates the median value; edges of boxes, upper and lower quartiles; and whiskers, the minimum and maximum values. NS indicates not significant. ^a^*P* < .001. ^b^*P* < .01. ^c^*P* < .05.

Adolescent internalizing and externalizing scores were the lowest in the low-adversity followed by medium-adversity, MD, and high-adversity profiles (Figure 2) (internalizing: *F*_(3,3333)_ = 37.84; *P* < .001; externalizing: *F*_(3,3332)_ = 60.04; *P* < .001). Internalizing symptoms did not differ between the MD and high-adversity profiles (mean [SD] score for MD profile: 0.221 [0.802]; for high-adversity profile: 0.335 [0.804]; mean difference, 0.11; 95% CI, −0.03 to 0.26; *P* = .18) (eTable 13 in Supplement 1), despite MD and medium-adversity profiles sharing the most similarities in adversity levels. Mean differences in externalizing symptoms differed among all profiles (eg, medium- vs low-adversity profiles: mean difference, 0.25; 95% CI, 0.18-0.32; *P* < .001) (eTable 13 in Supplement 1). Findings remained after adjusting for sociodemographic covariates (eTable 14 in Supplement 1). Exploratory analyses stratified by sex found that the difference between MD and high-adversity profiles was not statistically significant among female participants, but it was significant among male participants (eTable 18 and eFigure 7 in Supplement 1).

### Profile-Specific Subgroup Comparison in Functional Network Connectivity

**Figure 3.**
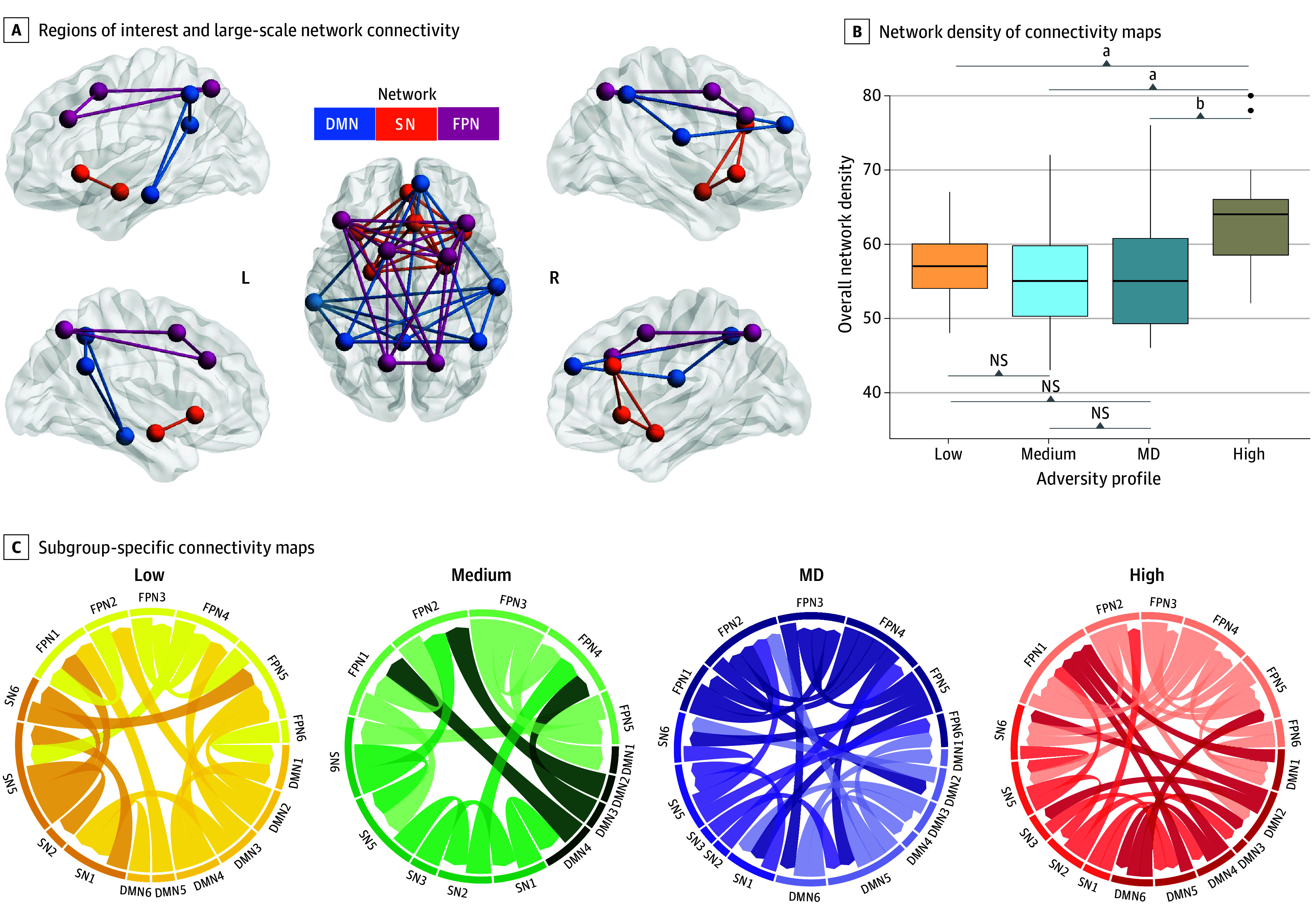
Brain Connectivity and Profile-Specific Networks A, The default mode network (DMN) included the bilateral inferior parietal lobule, posterior cingulate cortex, and medial temporal gyrus. The salience network (SN) included the bilateral insula, amygdala, and dorsal anterior cingulate cortex. The frontoparietal network (FPN) included the bilateral dorsolateral prefrontal cortex, anterior inferior parietal lobule, and posterior parietal cortex. B, Center line of boxplot indicates the median value; edges of boxes, upper and lower quartiles; and whiskers, the minimum and maximum values. C, Paths within each network map represent subgroup-specific connections. *P* values were adjusted for multiple comparisons. MD indicates maternal depression; NS, not significant. ^a^*P* < .001. ^b^*P* < .01.

**Figure 4.**
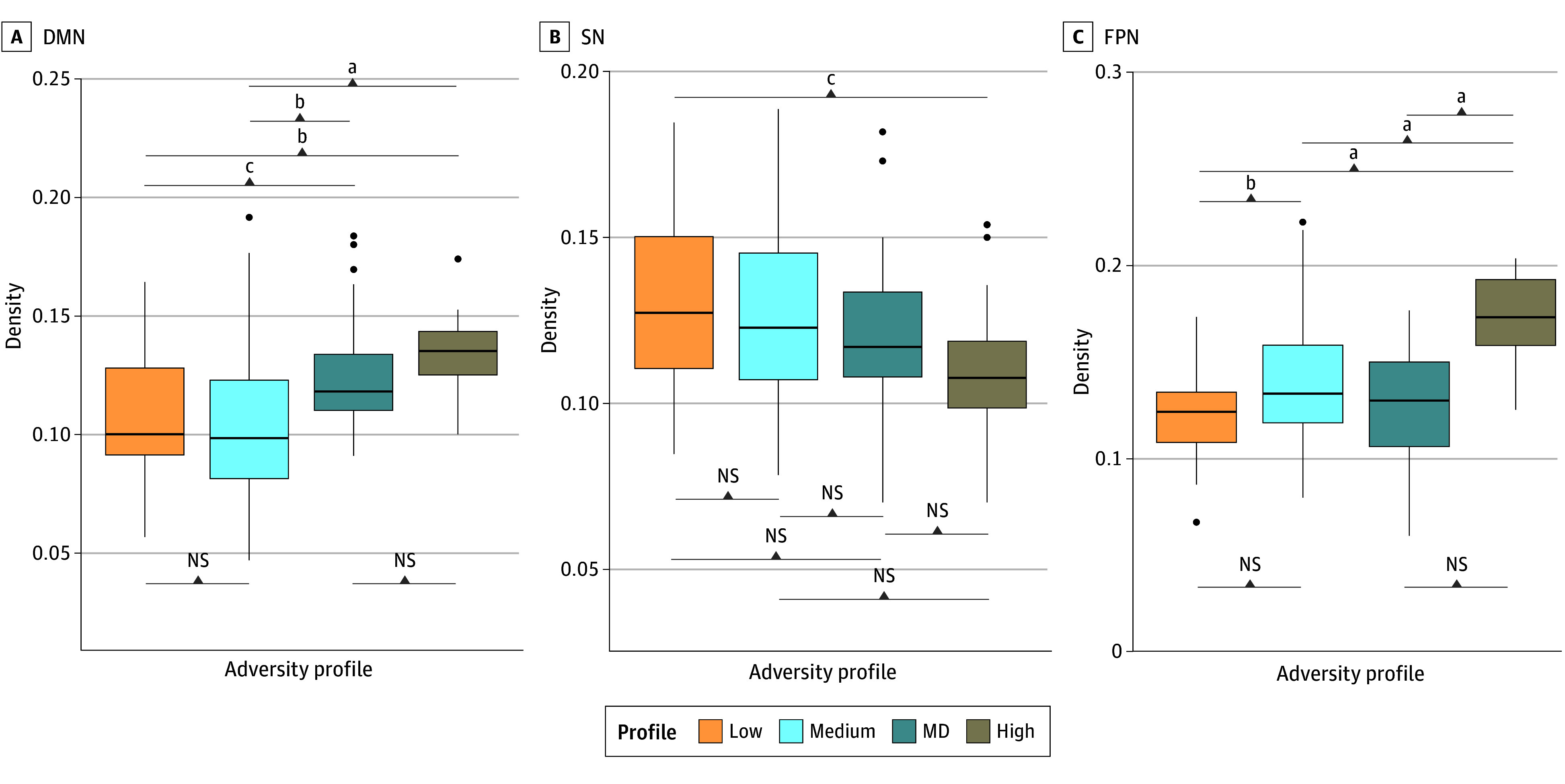
Boxplots Showing Differences in Subnetwork-Specific Connections Across Adversity Profiles DMN indicates default mode network; FPN, frontoparietal network; MD, maternal depression; NS, not significant; SN, salience network. *P* values shown were adjusted for multiple comparisons. ^a^*P* < .001. ^b^*P* < .01. ^c^*P* < .05.

Confirmatory subgrouping GIMME generated person-specific models with excellent fit (average indices: root mean square error of approximation, 0.06; standard root mean residual, 0.05; nonnormed fit index, 0.92; confirmatory fit index, 0.95). Group-level connections pertaining to individuals across all profiles were detected within the DMN, SN, and FPN (eFigure 5 in Supplement 1). Subgroup-level and individual-level connections specific to each profile were also identified across all networks, with more person-specific connections present for the high-adversity profile (Figure 3). There were also profile differences in both the overall density across the entire network and specific network densities. Overall network density differed among profiles (*F*_(3,163)_ = 10.65; *P* < .001) (Figure 3). Relative to the high-adversity profile, other adversity profiles showed lower density in the overall network (eTable 15 in Supplement 1). There were also differences in the specific network features and pairwise differences among adversity profiles (Figure 4). First, for the DMN, MD and high-adversity profiles showed higher density relative to the other profiles (*F*_(3,163)_ = 11.14; *P* < .001). The high-adversity profile also showed lower SN density compared with the low-adversity profile (mean difference, −0.2; 95% CI, −0.04 to −0.003;* P* *=* .03) (eTable 15 in Supplement 1) and the highest FPN density compared with other profiles (*F*_(3,163)_ = 18.96; *P* < .001) (eTable 15 in Supplement 1). These profile differences remained after adjusting for sociodemographic covariates (eTable 16 in Supplement 1). Moreover, these profile differences were observed using task-based functional networks but not resting-state networks, providing evidence for the specificity of these associations to emotion processes (eTable 17 and eFigure 6 in Supplement 1). There were no notable differences between female and male participants (eTable 19 in Supplement 1).

## Discussion

This study investigated associations among person-centered childhood adversity profiles, youth mental health, and emotion-related brain function within a population-based birth cohort. Four latent, multidomain childhood adversity profiles were identified: low-adversity, medium-adversity, MD, and high-adversity. Although individuals in the medium-adversity and MD profiles shared similar levels of overall exposure to adversity, the MD profile exhibited elevated internalizing symptoms, similar to the high-adversity profile. During an emotion task, youths with the MD and high-adversity profiles displayed the highest DMN density compared with those with the other 2 profiles. Additionally, those with the high-adversity profile exhibited attenuated SN density relative to the low-adversity profile and the highest FPN density relative to all other profiles.

The differences in symptomatic presentation among adversity profiles highlight the importance of clustered multidomain childhood adversity for adolescent mental health. Consistent with evidence that the accumulation of exposures to various risk factors could result in adverse health outcomes,^56^ the present study found that high exposure to adversity across multiple domains was associated with the highest mental health symptoms. Notably, a profile emerged with moderate levels of adversity and a high level of maternal depression (the MD profile). Despite similarities with the medium-adversity profile, youths with the MD profile showed mental health outcomes akin to those who were exposed to high levels of adversity across all domains (the high-adversity profile), especially for internalizing symptoms. This indicates a potentially influential role of MD in shaping youth mental health.

The intergenerational transmission of depression from mothers to children is widely recognized to involve both genetic and environmental mechanisms.^57,58,59^ Infants born to mothers with depression are at heightened risk of increased stress sensitivity and negative caregiving behaviors.^58^ In the present study, youths with the childhood adversity profile characterized by high MD had elevated levels of psychopathology, consistent with studies indicating strong links between maternal depression and child psychopathology. Moreover, these patterns were particularly important for female participants relative to male participants, consistent with previous work showing sex differences in stress-linked anxiety and depression.^58,60^

There were also profile-specific differences in brain function in networks key to mental health outcomes. Youths with the MD profile and those with the high-adversity profile exhibited similar patterns in the DMN during an emotion task, but not at rest, suggesting that these divergent network patterns were specific to affective conditions. Given that the DMN is typically deactivated during tasks,^61^ these findings suggest a more pronounced neural disengagement to emotional cues in youth who had high exposure to maternal depression and were exposed to many forms of adversity at a high level. Youths with the high-adversity profile also showed network differences across the SN and FPN compared with youth in other profiles. Whereas weak SN engagement has been attributed to disruptions in brain network communications,^31,62^ increased connectivity within the FPN could indicate a compensatory mechanism^63,64^ that is reflected in heightened cognitive control processes during emotion tasks. These findings suggest that high levels of exposures to a wide range of childhood adversity are associated with differences in neural network communications in critical regions of emotion regulation.

### Limitations

This study has limitations. First, there are likely many other important adversity exposures not measured here. Nonetheless, the study used information from multiple levels of risk factors across development, which may have captured much of the variance in the child’s adverse experiences. Second, many of the childhood adversity measures were parent-reported. Further research is needed to include data from other informants. Third, as this is not a genetically informed design, we are not able to disentangle genetic vs environmental influences, particularly in the associations between MD and youth outcomes. Fourth, the neuroimaging subsample is modestly sized, which precluded the examination of brain-behavior associations; thus, these results need to be reproduced in larger neuroimaging samples. Fifth, GIMME requires a priori specification of ROIs and networks; thus, these findings need to be examined across large-scale brain-wide networks. Sixth, no neuroimaging data were collected in childhood; thus, research is needed to examine the trajectories of these networks in relation to adversity exposure. Additionally, there are limitations inherent in the latent profile approach. LPA is unable to capture developmentally specific variations. Thus, although these results represent adversities across the first 9 years of age, they could not address the developmental specificity of adversity exposures. Moreover, while LPA can identify latent subgroups in specific samples, the generalizability of these profiles needs to be tested in additional cohorts; although notably, the latent profiles identified in the present investigation were modeled in a large representative sample, which serves to improve generalizability to the population.

## Conclusions

In a 15-year longitudinal study of a population-based birth-cohort sample, 4 profiles of childhood adversity with distinct associations with adolescent mental health and emotion-related brain function were identified. Adolescents exposed to high MD and high multidomain adversity in childhood were at the highest risk for psychopathology and had differential patterns across brain networks implicated in emotion processing relative to those with low- and medium-adversity profiles. To our knowledge, this study is the first to combine subtyping of adversity with individualized network estimation methods to parse heterogeneity both within the childhood adverse environment and subsequent brain networks in a longitudinal population-based sample. This study demonstrates the benefit of individual-oriented approaches in increasing the precision of neural mechanisms linked to adverse childhood experiences.

## Notes

Supplement 1.
eMethods.
**eTable 1. **Statistical Comparison Between the Full FFCWS and Included Samples **eTable 2. **Descriptives and Statistical Comparison Between Included and Neuroimaging Samples **eTable 3. **MNI Coordinates of Neural Regions of Interest (ROIs) **eTable 4. **Zero-Order Correlations Of Adversity Variables **eTable 5. **Model Fit Indices Between Latent Profile Classes **eTable 6. **Average Posterior Probabilities of Assigned Profile Membership (4-Class Model) **eTable 7. **Average Posterior Probabilities of the 3-Class and 5-Class Models **eTable 8. **Supplementary Latent Profile Analyses (4-Class Model) Leaving One Site Out **eTable 9. **Descriptives of Each Adversity Latent Profile in the Neuroimaging Subsample **eTable 10. **Mean and Standard Deviation of Adversity for Each **eTable 11. **Mean and Standard Deviation of Adversity in the Neuroimaging Subsample **eTable 12. **Pairwise Test Comparing Adversity Levels Among Latent Profiles **eTable 13. **Comparison of Youth Internalizing and Externalizing Among Adversity Profiles **eTable 14. **Comparison of Youth Internalizing and Externalizing Among Adversity Profiles, Adjusting for Covariates **eTable 15. **Comparison of Functional Connectivity Density Among Adversity Profiles **eTable 16. **Comparison of Functional Connectivity Density Among Profiles, Adjusting for Covariates **eTable 17. **Comparison of network Connectivity Metrics Estimated Using Neuroimaging Data During Emotional Faces Task vs Resting State Data **eTable 18. **Exploratory Analysis Comparing Youth Internalizing and Externalizing Among Adversity Profiles, Stratified by Sex **eTable 19. **Exploratory Analysis Comparing Functional Connectivity Density Among Adversity Profiles, Stratified by Sex **eFigure 1. **Exclusionary Criteria for the Neuroimaging Subsample **eFigure 2. **Internalizing Latent Factor Structure and Loadings **eFigure 3. **Externalizing Latent Factor Structure and Loadings **eFigure 4. **Prevalence of Adversity Indicators for the 4-Class Model Within the Neuroimaging Subsample **eFigure 5. **Confirmatory Subgrouping Group Iterative Multiple Model Estimation Network Plots for Each Adversity Profile **eFigure 6. **Boxplot Showing Network Density Estimated Using Resting-State Functional Neuroimaging Data **eFigure 7. **Youth Mental Health, Stratified by Sex
eReferences.

Supplement 2.
Data Sharing Statement
