# Supplementary material for: Latent Profiles of Childhood Adversity, Adolescent Mental Health, and Neural Network Connectivity
Source: JAMA Netw Open. 2024 Aug 28;7(8):e2430711. doi: 10.1001/jamanetworkopen.2024.30711 (PMC11358864; doi:10.1001/jamanetworkopen.2024.30711)
Supplement: Supplement 1. — eMethods. eTable 1. Statistical Comparison Between the Full FFCWS and Included Samples eTable 2. Descriptives and Statistical Comparison Between Included and Neuroimaging Samples eTable 3. MNI Coordinates of Neural Regions of Interest (ROIs) eTable 4. Zero-Order Correlations Of Adversity Variables eTable 5. Model Fit Indices Between Latent Profile Classes eTable 6. Average Posterior Probabilities of Assigned Profile Membership (4-Class Model) eTable 7. Average Posterior Probabilities of the 3-Class and 5-Class Models eTable 8. Supplementary Latent Profile Analyses (4-Class Model) Leaving One Site Out eTable 9. Descriptives of Each Adversity Latent Profile in the Neuroimaging Subsample eTable 10. Mean and Standard Deviation of Adversity for Each eTable 11. Mean and Standard Deviation of Adversity in the Neuroimaging Subsample eTable 12. Pairwise Test Comparing Adversity Levels Among Latent Profiles eTable 13. Comparison of Youth Internalizing and Externalizing Among Adversity Profiles eTable 14. Comparison of Youth Internalizing and Externalizing Among Adversity Profiles, Adjusting for Covariates eTable 15. Comparison of Functional Connectivity Density Among Adversity Profiles eTable 16. Comparison of Functional Connectivity Density Among Profiles, Adjusting for Covariates eTable 17. Comparison of network Connectivity Metrics Estimated Using Neuroimaging Data During Emotional Faces Task vs Resting State Data eTable 18. Exploratory Analysis Comparing Youth Internalizing and Externalizing Among Adversity Profiles, Stratified by Sex eTable 19. Exploratory Analysis Comparing Functional Connectivity Density Among Adversity Profiles, Stratified by Sex eFigure 1. Exclusionary Criteria for the Neuroimaging Subsample eFigure 2. Internalizing Latent Factor Structure and Loadings eFigure 3. Externalizing Latent Factor Structure and Loadings eFigure 4. Prevalence of Adversity Indicators for the 4-Class Model Within the Neuroimaging Subsample eFigure 5. Confirmatory Subgrouping G [file jamanetwopen-e2430711-s001.pdf]

Supplemental Online Content

Hardi FA, Beltz AM, McLoyd V, et al. Latent profiles of childhood adversity, adolescent mental health, and neural network connectivity. *JAMA Netw Open.* 2024;7(8):e2430711. doi:10.1001/jamanetworkopen.2024.30711

eMethods.

eTable 1. Statistical Comparison Between the Full FFCWS and Included Samples

eTable 2. Descriptives and Statistical Comparison Between Included and Neuroimaging Samples

eTable 3. MNI Coordinates of Neural Regions of Interest (ROIs)

eTable 4. Zero-Order Correlations Of Adversity Variables

eTable 5. Model Fit Indices Between Latent Profile Classes

eTable 6. Average Posterior Probabilities of Assigned Profile Membership (4-Class Model)

eTable 7. Average Posterior Probabilities of the 3-Class and 5-Class Models

eTable 8. Supplementary Latent Profile Analyses (4-Class Model) Leaving One Site Out

eTable 9. Descriptives of Each Adversity Latent Profile in the Neuroimaging Subsample

eTable 10. Mean and Standard Deviation of Adversity for Each

eTable 11. Mean and Standard Deviation of Adversity in the Neuroimaging Subsample

eTable 12. Pairwise Test Comparing Adversity Levels Among Latent Profiles

eTable 13. Comparison of Youth Internalizing and Externalizing Among Adversity Profiles

eTable 14. Comparison of Youth Internalizing and Externalizing Among Adversity Profiles, Adjusting for Covariates

eTable 15. Comparison of Functional Connectivity Density Among Adversity Profiles

eTable 16. Comparison of Functional Connectivity Density Among Profiles, Adjusting for Covariates

eTable 17. Comparison of network Connectivity Metrics Estimated Using Neuroimaging Data During Emotional Faces Task vs Resting State Data

eTable 18. Exploratory Analysis Comparing Youth Internalizing and Externalizing Among Adversity Profiles, Stratified by Sex

eTable 19. Exploratory Analysis Comparing Functional Connectivity Density Among Adversity Profiles, Stratified by Sex

eFigure 1. Exclusionary Criteria for the Neuroimaging Subsample

eFigure 2. Internalizing Latent Factor Structure and Loadings

eFigure 3. Externalizing Latent Factor Structure and Loadings

eFigure 4. Prevalence of Adversity Indicators for the 4-Class Model Within the Neuroimaging Subsample

eFigure 5. Confirmatory Subgrouping Group Iterative Multiple Model Estimation Network Plots for Each Adversity Profile

© 2024 Hardi FA et al. *JAMA Network Open.*

eFigure 6. Boxplot Showing Network Density Estimated Using Resting-State Functional Neuroimaging Data
eFigure 7. Youth Mental Health, Stratified by Sex
eReferences.

This supplemental material has been provided by the authors to give readers additional information about their work.

© 2024 Hardi FA et al. *JAMA Network Open.*

eMethods

Childhood adversity measures

Ten variables were examined as indicators of childhood adversity spanning across four waves (ages 1, 3, 5, and 9) capturing information about the child's environment from birth to age 9. The use of these variables was justified in past publications[1–5] as available constructs representing salient childhood adversities in this sample. These variables provide information on child maltreatment (physical abuse, emotional abuse, and neglect), intimate partner violence (IPV), maternal depression, parental stress, residential moves, and neighborhood adversities (lack of cohesion, lack of social control, neighborhood violence). Each type of adversity is associated with a large body of literature connecting it to negative outcomes, particularly internalizing and externalizing psychopathology.

Childhood maltreatment data were collected when the child was 3, 5, and 9 years old. Each maltreatment type (physical abuse, emotional abuse, and neglect) was measured by separate subscales in the Parent-Child Conflict Tactics Scale.[6] Emotional abuse was parent-reported using the 5-item psychological aggression subscale capturing past year frequency that the primary caregiver reported to have engaged in behaviors such as "shouted, yelled, or screamed at" or "swore or cursed at" child (0 = did not happen, 1 = has happened one or more times).[6] Physical abuse was parent-reported using the 5-item physical assault subscale capturing past year frequency that the primary caregiver reported to have engaged in behaviors such as "spanked [child] on the bottom with their bare hand" or "hit [child] on the bottom with something like a belt, hairbrush, a stick or some other hard object." (0 = did not happen, 1 = has happened one or more times).[6] Neglect was parent-reported using the 5-item neglect subscale

© 2024 Hardi FA et al. *JAMA Network Open.*

capturing past year frequency that primary caregiver reported to have engaged in behaviors such as "had to leave their child home alone, even when they thought some adult should be with him/her" or "was not able to make sure their child got to a doctor or hospital when he/she needed it." (0 = did not happen, 1 = has happened one or more times).[6] Average scores for each subscale across all waves were computed to represent the extent of childhood physical abuse, emotional abuse, and neglect.

Intimate partner violence (IPV) data were collected when the child was 1, 3, 5, and 9 years old using parent-reported 6-item questions on physical, emotional, or sexual intimate partner violence such as "how often does father slap or kick you?" or "how often does father try to isolate you from friends/family?" (0 = never, 1 = sometimes, 3 = often) perpetrated by the child's father or parent's romantic partner. These items were selected based on a previous study on adverse childhood experiences in this sample.[7] In cases where the mother was no longer in a relationship with the child's biological father during the data collection wave, the mother reported information about her current partner. An average score across all waves was computed to represent IPV.

Maternal depression was measured using self-reported data on the Composite International Diagnostic Interview – Short Form (CIDI-SF)[8] when the child was 1, 3, 5, 9 years old. The CIDI-SF, consistent with the Diagnostic and Statistical Manual of Mental Disorders – Fourth Edition,[9] included questions on the mother's feelings of depressed mood or anhedonia (loss of pleasure or interest in activities that they usually found enjoyable) in the past year that lasted two weeks or more (1 = yes, 0 = no). If so, they were asked more detailed questions about losing interest, tiredness, changes in weight, sleep, concentration, worthlessness, and any suicidal ideation. Diagnostic criteria were met if the mother endorsed depressed mood or anhedonia

© 2024 Hardi FA et al. *JAMA Network Open.*

lasting at least half of the day nearly every day and two or more additional symptoms. An average score across all waves was computed to represent maternal depression across childhood.

Parental stress was measured when the child was 1, 3, 5, and 9 years old using a 4-item parent-reported questions adapted from the Child Development Supplement of the Panel of Study of Income Dynamics,[10] with example questions such as “I often feel tired, worn out, or exhausted from raising a family” and “I feel trapped by my responsibilities as a parent.” (0 = strongly disagree, 1 = somewhat disagree, 2 = somewhat agree, 3 = strongly agree). Several items for this scale were taken from the Parent Stress Inventory,[11] which measures stress triggered by changes in employment, income or other factors. An average score across all waves was computed to represent parental stress.

The frequency of residential moves or household instability was parent-reported when the child was 1, 3, 5, and 9 years old to capture changes occurring in between waves (i.e., between ages 0-1, 1-3, 3-5, and 5-9). At each wave, mothers or primary caregivers provided answers on whether the family has moved since the prior wave, and if yes, how many times. An average score across all waves was computed to represent residential moves across childhood.

Three neighborhood factors (lack of community cohesion, lack of social control, and neighborhood violence) were reported when the child was 3, 5, and 9 years old. Lack of neighborhood cohesion was measured using parent-reported reverse-coded 4-item questions taken from the Social Cohesion and Trust Scale,[12,13] with example questions such as “people around here are willing to help their neighbors” and “this is a close-knit neighborhood” (0 = strongly agree, 1 = agree, 2 = disagree, 3 = strongly disagree). Lack of neighborhood social control was measured using reverse-coded 5-item questions taken from the Informal Social Control Scale[12,13] such as “how likely neighbors intervene if children skipping school and

© 2024 Hardi FA et al. *JAMA Network Open.*

hanging on street?" and "how likely neighbors intervene if fight broke out in front of the house?" (0 = very likely, 1 = somewhat, 2 = not very unlikely, 3 = very unlikely). Neighborhood violence was measured using 3 parent-reported items such as "in the past year, how often did you see person get hit, slapped, punched?" and "in the past year, how often did you see person attacked with weapon?" (0 = never, 1 = once, 2 = 2-3 times, 3 = 4-10 times, 4 = more than 10 times) based on prior investigations.[14] Average scores across all waves for each construct were computed to represent the lack of community cohesion, lack of social control, and neighborhood violence during childhood.

Youth internalizing and externalizing symptoms

Internalizing symptoms were measured as a multi-informant latent factor comprised of all available FFCWS measures of internalizing symptoms at age 15: parent-reported internalizing scale (i.e., anxious/depressed and withdrawn items) from the Child Behavioral Checklist 6-18 (CBCL);[15] youth-reported items from the Brief Symptom Inventory 18 (BSI-18);[16] and youth-reported items from the Center for Epidemiologic Studies Depression Scale (CES-D).[17] The CBCL is comprised of 8 questions (6 anxious/depressed items and 2 withdrawn items) (0 = not true, 1 = sometimes true, 2 = often true), and higher scores indicate greater youth internalizing symptoms. The BSI-18 contains 6 questions from the anxiety subscale (0 = strongly disagree, 1 = somewhat disagree, 2 = somewhat agree, 3 = strongly agree), and higher scores indicate greater anxiety symptoms. The CES-D contains 4 questions (0 = strongly disagree, 1 = somewhat disagree, 2 = somewhat agree, 3 = strongly agree), and higher scores indicate greater youth depressive symptoms. Confirmatory factor analysis was conducted using MPlus v8.8[18] with WLSMV estimator to account for categorical variables. Each question was loaded onto three

© 2024 Hardi FA et al. *JAMA Network Open.*

latent factors reflecting the measures (CBCL, BSI-18, CES-D), which were then loaded onto a higher-order latent factor of overall internalizing symptoms. Model fit indices indicate adequate model fit (CFI = .931, TLI = .921, RMSEA = .065, SRMR = .075)[19] (eFigure 2). Internalizing factor scores were then extracted as individual scores for further analysis.

Externalizing behaviors were measured as a multi-informant latent factor comprised of all available FFCWS measures of externalizing behavior at age 15: parent-reported externalizing scale (i.e., aggressive and rule-breaking items) from the Child Behavioral Checklist 6-18 (CBCL);[15] youth-reported items from the Delinquency scale adopted from the National Longitudinal Study of Adolescent Health (Add Health);[20] and youth-reported substance use. The parent-reported CBCL items comprised of 19 questions (10 aggressive behavior items and 9 rule-breaking behavior items) (0 = not true, 1 = sometimes true, 2 = often true), and higher scores indicate greater youth externalizing symptoms. Youth-reported delinquency was measured by 13 questions (0 = never, 1 = sometimes, 2 = often), and higher scores indicate greater youth delinquent behavior. Substance use was measured using 5 binary questions (0 = no, 1 = yes) capturing alcohol use (more than 2 drinks without parents), tobacco, and other substances (marijuana, illicit drugs or nonmedical use of prescription drugs). Confirmatory factor analysis was conducted using Mplus v8.8[18] with WLSMV estimator to account for categorical variables. Each question was loaded onto three latent factors reflecting the measures (CBCL, Delinquency, Substance), which were then loaded onto a higher-order latent factor of overall externalizing behavior. Model fit indices indicate excellent model fit (CFI = .955, TLI = .952, RMSEA = .031, SRMR = .092)[19] (eFigure 3). Externalizing factor scores were then extracted as individual participant scores for further analysis.

© 2024 Hardi FA et al. *JAMA Network Open.*

Sociodemographic covariates

The following covariates were included in sensitivity analyses: racial and ethnic identity, parental marital status, and household income. Two additional covariates were included in the neuroimaging subsample analysis: the age of the youth during the neuroimaging scan and in-scanner motion. Racial and ethnic identity was included to account for unequal exposures to experiences of race-related adversity such as discrimination and structural racism and was youth-reported at age 15 (Black/African American, non-Hispanic; Hispanic, all races; Multi-racial, non-Hispanic; White, non-Hispanic; Other, non-Hispanic). For those youth who did not participate in the wave 15 data collection, the mother's self-report of race and ethnicity at baseline was used to describe the sample. There was a 91% coherence between youth-reported race and ethnicity and mother's self-reported race and ethnicity for those individuals with both youth-reported and mother-reported race and ethnicity data. As the group with the highest prevalence in the sample, the Black group was used as the reference group in all statistical models. There was no additional information on the breakdown of the Other subcategory. The majority of the subcategory comprised of Asian and Pacific Islanders, but the specific breakdown is not available to the public as it may reveal identifying information about the participants. Parental marital status was included to account for FFCWS sampling strategy,[21] and was parent-reported when each child was age 1 (0 = Unmarried, 1 = Married). Household income was included to account for differences in family socioeconomic resources and was measured by poverty ratio (ratio of total household income to the official poverty thresholds designated by the U.S. Census Bureau), which was parent-reported at age 1 (higher poverty ratio indicated higher socioeconomic status). Youth age was included to account for differences in stages of normative brain development and was computed using the youth-reported date of birth at age 15. In-scanner

© 2024 Hardi FA et al. *JAMA Network Open.*

motion was measured using the framewise displacement metric computed in FSL by averaging the differences in rotation and translation parameters,[22] and was included to ensure that results were robust after adjustment for motion differences. Analysis of covariance (ANCOVA) models were first tested to examine differences in network connectivity metrics among profiles, accounting for all covariates. Pairwise multiple comparisons were then conducted with adjustment for multiple comparisons using the Tukey-Kramer test.

Procedures and robustness checks for LPA

Latent profile analysis was conducted using Mplus v8.8.[18] Latent class models were estimated by adding classes in consecutive order; starting with a two-class model. Classes were added iteratively until the final model was identified. In all models, proportional covariance structure was used to assist in convergence for complex models.[23] Here, covariance in a class was freely estimated and used as a referent, resulting in equal correlation matrices without constrained homogeneity of covariance structures across classes.[23–26] Classes were initially fitted using 500 random starts with 20 iterations, and then repeated with 1000 and, subsequently, 2000 starting values to ensure that the results reflect a global maximum.[27]

Multiple model fit indices and classification characteristics (log-likelihood (LL), Akaike Information Criteria (AIC), Bayesian Information Criteria (BIC), adjusted BIC (ABIC), Lo-Mendell-Rubin Adjusted Likelihood Ratio test (LMR), Entropy, average latent class posterior probabilities, class sizes) were used to determine model selection.[27–29] LL represents the goodness-of-fit of the model, with higher values indicating a better fit. AIC,[30] BIC,[31] and ABIC[32] are statistical information criteria, with lower values indicating a better model fit. LMR[33] is a test comparing the specified model *k* with *k*-1 class (model with one fewer class), and assesses if

there are statistically meaningful improvements in model fit with the addition of one class. Classifications diagnostics were also examined for class selection.[34] Entropy is a measure of class separation and assesses the classification accuracy,[35] where high values (1.0 being the maximum, .80 to be acceptable)[35] indicate high separation among classes. The average posterior probabilities represent the certainty of latent profile assignment, whereby high classification quality is achieved when the diagonal values are high (as close to the maximum value of 1.0) and off-diagonal values are low (as close to the minimum value of 0).[36] Finally, class sizes were examined to ensure that no class has fewer than 50 individuals or 5% of the sample, which is prone to model misspecifications.[27,28]

Loglikelihood was replicated for all fitted classes across different starting values with the exception of the 6-class model. Multiple model fit indices improved with a greater number of classes (i.e., increases in LL and decreases in AIC, BIC, and ABIC values with more fitted classes) until the 6-class model, for which poorer model fit and classification were examined across multiple parameters (eTable 5), suggesting that a 6-class model may be too high in complexity for the present data. Thus, no additional models beyond the 6-class model were estimated. The model fit and classification indices were then compared among the remaining estimated classes for the final model selection.

Results demonstrate a 4-class model to be the best-fitting solution with the greatest parsimony. Specifically, the 4-class model showed improvements across all model fit indices (highest LL and lowest AIC, BIC, and ABIC values) compared to the 2- and 3-class models, and the highest classification accuracy (highest Entropy and average posterior probabilities) among all other class models (eTable 5). In the four-class model, average posterior probabilities ranged from .874 to .919 (eTable 6), with approximately 11.8% of the sample with values below .70.

© 2024 Hardi FA et al. *JAMA Network Open.*

These statistics indicate a greater classification quality for the 4-class model compared to the 3-class and the 5-class models (eTable 7), for which several diagonal class posterior probabilities fall below the optimal range of .80 to .90,[28,36] and contained a greater proportion of individuals with low posterior probability of below .70[37] (3-class: 12.9%; 5-class: 19.8%).

To determine the internal consistency and robustness of the selected final model, LPA with the fitted 4-class model was repeated for a total of 20 supplementary analyses, leaving out one site (i.e., sample city)[21] at a time. The consistency of model fit parameters and prevalence of the resulting class memberships were then examined across these analyses. Results demonstrated convergence across these separate supplementary analyses of the selected latent profile 4-class model (eTable 8). The resulting profile membership of each individual in the 4-class model was then extracted for additional analysis.

Neuroimaging data acquisition and preprocessing

MRI data were acquired using a 3T GEDiscovery MR750 scanner with an 8-channel head coil. Head padding and instructions limited movement. T1-weighted gradient echo images were first captured (TR=12ms, TE=5ms, TI=500ms, flip angle=15°, FOV=26cm, slice thickness=1.44mm, 256x192 matrix, 110 slices). fMRI T2*-weighted blood oxygenation level dependent (BOLD) images were then captured using reverse spiral sequence[38] of 40 contiguous axial 3mm slices (TR = 2000ms, TE = 30ms, flip angle = 90°, FOV = 22cm, voxel size = 3.44mm x 3.44mm x 3mm, ascending acquisition, parallel to AC-PC line).

Task-based functional neuroimaging (fMRI) data were collected using an event-related emotion (faces) task (see [39] for a visual representation of task paradigm design). Participants were shown a series of emotional faces[40] and indicated if they were viewing a female or male

© 2024 Hardi FA et al. *JAMA Network Open.*

face. Gender (female, male), race (Black/African American, White), and emotion (fearful, happy, sad, neutral, angry) of the actor were counterbalanced and randomly presented across 100 trials. Each trial consisted of a fixation cross (500ms) followed by 250ms of an emotional face, then 1500ms of blank screen during which participants were expected to respond using a button press. Functional data from each participant across all trials of the emotion task (without any contrasting across emotion conditions) were extracted for subsequent processing. Resting-state neuroimaging data were collected while participants were awake and passively viewing a fixation cross.

Identical preprocessing steps were applied to both task-fMRI and resting-state fMRI data. Anatomical images were first skull-stripped (f=.25) using Brain Extraction Tool (BET) in FSL version 6.0[41] and segmented into gray matter, white matter, and cerebrospinal fluid using FSL FAST. After large temporal spikes in the k-space functional data (>2 *SD*) were removed, field maps were corrected and functional images were reconstructed using MATLAB. Noise from cardiac and respiratory motion was removed using RETROICOR and slice-timing correction using SPM8 (Wellcome Department of Cognitive Neurology, London, UK; http://www.fil.ion.ucl.ac.uk). Moreover, the first ten volumes of functional data were removed to ensure the stability of signal intensity. Following these steps, the functional data were further preprocessed using FSL fMRI Expert Analysis Tool (FEAT). Functional images were skull-stripped and spatially smoothed using FSL FMRIB's Automated Segmentation Tool,[42] and registered to subject-specific previously skull-stripped and segmented anatomical images. Motion correction was performed using MCFLIRT and spatial smoothing using a Gaussian kernel of FWHM 6.0mm was applied. The grand-mean intensity of the entire 4D dataset was normalized by a single multiplicative factor and FSL motion outliers were run to extract

© 2024 Hardi FA et al. *JAMA Network Open.*

framewise displacement motion parameters.[22] ICA-AROMA was used to remove motion-related artifacts in the data; nuisance signal derived from white matter and cerebrospinal fluid was regressed out; and data with signal below 0.01Hz were then high-pass filtered. These preprocessing steps were applied using detailed scripts[43] similar to prior work.[1,39]

Functional connectivity across neural networks estimation

ROI selection and data extraction

The present investigation focused on eighteen bilateral regions that represent the tripartite network:[26] Default Mode Network (DMN), Salience Network (SN), Frontopariental Network (FPN) (eTable 3). The DMN extends across the lateral parietal, posterior cingulate, and medial temporal cortices.[44] It is often linked to introspective self-referential mental processes and is conventionally believed to deactivate during task-oriented engagement.[45] The SN includes the anterior insula, cingulate cortex, and amygdala, and plays a central role in detecting important environmental cues[46] and facilitating bottom-up signals to other networks.[47] The FPN, which encompasses the inferior lobule, dorsolateral prefrontal, and posterior parietal cortices, is implicated in cognitive control and goal-directed processes.[48]

Consistent with our previous investigations,[1,39] ROI coordinates were extracted from NeuroSynth,[49] a meta-analytic tool that combines results from published neuroimaging articles using an automated parser. Specific ROI names (i.e., “Default Mode”, “Salience”, “Frontoparietal”) were used as keywords to search for peak activity on the NeuroSynth website, and corresponding association maps were then downloaded. Voxel coordinates from downloaded images were subsequently extracted using FSL and then utilized to create an ROI 6.5mm-

diameter sphere using *fslmaths*.[1] The ROIs for DMN and SN in this study were consistent with a previous investigation,[1] and three additional nodes were selected to represent the FPN.

Confirmatory Subgrouping Group Iterated Multiple Model Estimation (GIMME)

Confirmatory Subgrouping GIMME[50] is an extension of GIMME,[51] a functional connectivity analysis method that iteratively fits unified structural equation models to arrive at person-specific networks that contain group-, subgroup-, and individual-level connections. GIMME estimates both directed contemporaneous (occurring at the same time or functional volume) and lagged (occurring at a different time or functional volume) connections among *a priori* regions of interest (ROIs). GIMME has been validated in multiple large-scale simulations to outperform 38 other commonly-used approaches in estimating connectivity maps among neural nodes,[51] and has been discussed in over 400 scientific articles.[51,52] GIMME begins search for group model with autoregressive paths freed for estimation. GIMME first estimates connections among preselected brain ROIs that pertain to at least 75% of the entire sample if the connections significantly improve individual model fit (as assessed by Lagrange Multiplier tests).[53] In the Confirmatory Subgrouping extension,[50] subgroup-specific connections are then estimated for individuals in each prespecified subgroup if the connections significantly improve model fit for at least 51% of individuals within each subgroup. Finally, individual-level connections that are specific to each person in the sample are estimated until the connectivity model fits the observed data for each individual well, according to traditional model fit indices. Contemporaneous connections estimated using GIMME were then extracted to compute network density measures for further analyses, consistent with previous investigations.[1,39]

© 2024 Hardi FA et al. *JAMA Network Open.*

Analyses comparing functional connectivity networks during emotion task vs non-task

There were differential patterns of resting-state network connectivity among adversity profiles compared to task-based network connectivity (eTable 17). Repeated measures ANOVAs were conducted with Greenhouse–Geisser correction to examine the differences between scan type (task vs. rest) in predicting network density (DMN, SN, FPN). Results demonstrated that task-based network connectivity significantly differed from network connectivity during the resting-state (eTable 17). Results from repeated-measure ANOVA comparing task-based from resting-state network connectivity found differences between scan type and by profiles (eTable 17). In particular, there were significant differences between scan type within person for overall network density ($F(1,150)=0.78$, ges=.092, $P<.001$) and SN density ($F(1,150)=9.71$, ges=.026, $P=.001$). Moreover, there were significant scan types by profile differences. Specifically, there were differences between task-based and resting-state data network connectivity in the DMN for low and medium-adversity profiles ($F(3,150)=7.52$, ges=0.63, $P<.001$); SN for maternal depression and high-adversity profiles ($F(3,150)=5.22$, ges=0.42, $P=.001$); and FPN for low-adversity and high-adversity profiles ($F(3,150)=16.56$, ges=.133, $P<.001$) (eTable 17).

Exploratory analysis examining differences among adversity profiles, stratified by sex

In exploratory analyses, sex was accounted for as a biological variable by separately examining the mean differences in mental health outcomes and metrics of functional connectivity networks among adversity profiles for males and females. Sex was considered as a biological factor assigned at birth, and was mother-reported at child birth (baseline wave) as “Male” or “Female”.

© 2024 Hardi FA et al. *JAMA Network Open.*

Findings are reported on eTable 18 and eTable 19. For youth internalizing and externalizing outcomes, similar patterns to the analysis with the entire sample were observed. Youth internalizing and externalizing outcomes increased from Low-adversity to Medium-adversity, MD, High-adversity profiles. For females, internalizing and externalizing symptoms do not differ between the MD and High-adversity profiles; whereas for males, internalizing and externalizing symptoms do not differ between the Medium-adversity and the MD profiles (eFigure 8; eTable 18). There were no notable sex differences between male and female groups in stratified analyses examining mean differences in brain network metrics (eTable 19).

© 2024 Hardi FA et al. *JAMA Network Open.*

eTable 1. Statistical Comparison Between the Full FFCWS and Included Samples

	**FFCWS sample (n = 4,898)**	**Included sample (n = 4,210)**	**Test**
Racial and ethnic identity *No.* (%)	Black non-Hispanic = 2284 (47%)<br>Hispanic = 1364 (28%)<br>Multiracial non-Hispanic = 175 (4%)<br>White non-Hispanic = 903 (18%)<br>Other = 167 (3%)	Black non-Hispanic = 1959 (47%)<br>Hispanic = 1169 (28%)<br>Multiracial non-Hispanic = 156 (4%)<br>White non-Hispanic = 786 (19%)<br>Other = 136 (3%)	$\chi^2(4)$ = 0.40, *P* = .98
Child sex *No.* (%)	Female = 2,568 (52%)<br>Male = 2,329 (48%)	Female = 1,999 (48%)<br>Male = 2,211 (53%)	$\chi^2(1)$ = .003, *P* = .96
Parental marital status *No.* (%)	Married = 1,187 (24%)<br>Unmarried = 3,710 (76%)	Married = 1,068 (25%)<br>Unmarried = 3,142 (75%)	$\chi^2(1)$ = 1.49, *P* = .22
Poverty ratio *M* (*SD*)	2.22 (2.41)	2.30 (2.47)	t(8838.4) = -1.59, *P* = .11
Child birth city *No.* (%)	Oakland, CA = 330 (7%)<br>Austin, TX = 326 (7%)<br>Baltimore, MD = 338 (9%)<br>Detroit, MI = 327 (7%)<br>Newark, NJ = 342 (7%)<br>Philadelphia, PA = 337 (7%)<br>Richmond, VA = 327 (7%)<br>Corpus Christi, TX = 331 (7%)<br>Indianapolis, IN = 325 (7%)<br>Milwaukee, WI = 348 (7%)<br>New York, NY = 384 (8%)<br>San Jose, CA = 326 (7%)<br>Boston, MA = 99 (2%)<br>Nashville, TN = 102 (2 %)<br>Chicago, IL = 155 (3%)<br>Jacksonville, FL = 100 (2%)<br>Toledo, OH = 101 (2%)<br>San Antonio, TX = 100 (2%)<br>Pittsburgh, PA = 100 (2%)<br>Norfolk, VA = 99 (2%)	Oakland, CA = 281 (7%)<br>Austin, TX = 282 (7%)<br>Baltimore, MD = 294 (7%)<br>Detroit, MI = 283 (7%)<br>Newark, NJ = 274 (7%)<br>Philadelphia, PA = 300 (7%)<br>Richmond, VA = 267 (6%)<br>Corpus Christi, TX = 296 (7%)<br>Indianapolis, IN = 286 (7%)<br>Milwaukee, WI = 312 (7%)<br>New York, NY = 312 (7%)<br>San Jose, CA = 270 (6%)<br>Boston, MA = 90 (2%)<br>Nashville, TN = 86 (2%)<br>Chicago, IL = 136 (3%)<br>Jacksonville, FL = 88 (2%)<br>Toledo, OH = 89 (2%)<br>San Antonio, TX = 88 (2%)<br>Pittsburgh, PA = 91 (2%)<br>Norfolk, VA = 85 (2%)	$\chi^2(19)$ = 3.19, *P* > .99

Note. Unknown group was omitted in statistical comparisons; Poverty ratio represents a ratio of total household income to the official poverty threshold at baseline (child birth), and higher values represent higher socioeconomic status. More information about the Other race and ethnicity category is not publicly available.

© 2024 Hardi FA et al. *JAMA Network Open.*

eTable 2. Descriptives and Statistical Comparison Between Included and Neuroimaging Samples

	**Included FFCWS sample (*n* = 4,210)**	**Neuroimaging subsample (*n* = 167)**	**Comparison**
Racial and ethnic identity *No.* (%)	Black non-Hispanic = 1959 (47%)<br>Hispanic = 1169 (28%)<br>Multiracial non-Hispanic = 156 (4%)<br>White non-Hispanic = 786 (19%)<br>Other = 136 (3%)	Black non-Hispanic = 128 (77%)<br>Hispanic = 11 (7%)<br>Multiracial non-Hispanic = 4 (2%)<br>White non-Hispanic = 20 (12%)<br>Other = 4 (2%)	$\chi^2(4)$ = 62.24, $P < .001$
Child sex *No.* (%)	Male = 2,211 (53%)<br>Female = 1,999 (48%)	Male = 76 (46%)<br>Female = 91 (55%)	$\chi^2(1)$ = 2.89, $P = .09$
Parental marital status *No.* (%)	Married = 1,068 (25%)<br>Unmarried = 3,142 (75%)	Married = 37 (22%)<br>Unmarried = 130 (78%)	$\chi^2(1)$ = 0.72, $P = .40$
Poverty ratio *M* (*SD*)	2.30 (2.47)	M (SD) = 2.11 (2.31)	$t(181.31) = 1.07$, $P = .28$
Child birth city *No.* (%)	Oakland, CA = 281 (7%)<br>Austin, TX = 282 (7%)<br>Baltimore, MD = 294 (7%)<br>Detroit, MI = 283 (7%)<br>Newark, NJ = 274 (7%)<br>Philadelphia, PA = 300 (7%)<br>Richmond, VA = 267 (6%)<br>Corpus Christi, TX = 296 (7%)<br>Indianapolis, IN = 286 (7%)<br>Milwaukee, WI = 312 (7%)<br>New York, NY = 312 (7%)<br>San Jose, CA = 270 (6%)<br>Boston, MA = 90 (2%)<br>Nashville, TN = 86 (2%)<br>Chicago, IL = 136 (3%)<br>Jacksonville, FL = 88 (2%)<br>Toledo, OH = 89 (2%)<br>San Antonio, TX = 88 (2%)<br>Pittsburgh, PA = 91 (2%)<br>Norfolk, VA = 85 (2%)	Baltimore = 1 (0.6%)<br>Detroit = 113 (68%)<br>Indianapolis = 2 (1%)<br>Chicago = 24 (14%)<br>Toledo = 26 (16%)<br>Pittsburgh = 1 (0.6%)	$\chi^2(5)$ = 193.52, $P < .001$

Note. Unknown group was omitted in statistical comparisons; Poverty ratio represents a ratio of household income to the official poverty threshold at baseline, higher values represent higher socioeconomic status; Only six cities (neuroimaging subsample) were included in birth city comparisons. Information about the Other race and ethnicity category is not publicly available.

© 2024 Hardi FA et al. *JAMA Network Open.*

eTable 3. MNI Coordinates of Neural Regions of Interest (ROIs)

**Default Mode Network (DMN)**		
DMN_1	R. Inferior Parietal Lobule	46 -52 48
DMN_2	L. Inferior Parietal Lobule	-42 -52 48
DMN_3	R. Posterior Cingulate Cortex	8 -52 28
DMN_4	L. Posterior Cingulate Cortex	-4 -52 28
DMN_5	R. Medial Temporal Gyrus	58 -16 20
DMN_6	L. Medial Temporal Gyrus	-62 -26 -18
**Salience Network (SN)**		
SN_1	R. Insula	36 20 -4
SN_2	L. Insula	-34 20 -4
SN_3	R. Amygdala	24 -2 -16
SN_4	L. Amygdala	-24 -6 -16
SN_5	R. Dorsal Anterior Cingulate Cortex	4 26 28
SN_6	L. Dorsal Anterior Cingulate Cortex	0 46 6
**Fronto Parietal Network (FPN)**		
FPN_1	R. Dorsolateral Prefrontal Cortex	38 26 34
FPN_2	L. Dorsolateral Prefrontal Cortex	-44 28 32
FPN_3	R. Anterior Inferior Parietal Lobule	26 4 50
FPN_4	L. Anterior Inferior Parietal Lobule	-14 8 50
FPN_5	R. Posterior Parietal Cortex	18 -66 50
FPN_6	L. Posterior Parietal Cortex	-14 -66 52

© 2024 Hardi FA et al. *JAMA Network Open.*

eTable 4. Zero-Order Correlations Of Adversity Variables

**Variables (avg. 0-9yo)**	**1**	**2**	**3**	**4**	**5**	**6**	**7**	**8**	**9**
1. Physical abuse									
2. Emotional abuse	.64**								
3. Neglect	.27**	.22**							
5. Maternal depression	.22**	.13**	.19**						
4. Intimate partner violence	.10**	.05*	.14**	.19**					
6. Parental stress	.23**	.18**	.24**	.26**	.15**				
7. Residential moves	.14**	.12**	.09**	.20**	.06**	.08**			
8. Lack of community cohesion	.22**	.16**	.14**	.19**	.14**	.19**	.14**		
9. Lack of community control	.09**	.09**	.12**	.09**	.13**	.14**	.11**	.56**	
10. Neighborhood violence	.18**	.14**	.13**	.15**	.07**	.13**	.08**	.31**	.15**

*Note. * indicates p < .05. ** indicates p < .01.*

© 2024 Hardi FA et al. *JAMA Network Open.*

eTable 5. Model Fit Indices Between Latent Profile Classes

Model	Log-likelihood (LL) (df)	% reduction in LL	AIC	BIC	ABIC	Entropy
2-class	-45462.39 (32)	NA	90988.77	91191.82	91090.14	0.78
3-class	-44309.84 (44)	2.54***	88707.68	88986.87	88847.06	0.76
4-class	-43538.83 (56)	1.74***	87189.66	87544.99	87367.05	0.82
5-class	-43033.89 (68)	1.16***	86203.79	86635.26	86419.19	0.79
6-class	-48469.01 (80)	-12.63	97098.02	97605.64	97351.43	0.78

*Note. ***p<.001 in likelihood ratio test. AIC indicates Akaike Information Criteria. BIC indicates Bayesian Information Criteria. ABIC indicates adjusted BIC.*

© 2024 Hardi FA et al. *JAMA Network Open.*

eTable 6. Average Posterior Probabilities of Assigned Profile Membership (4-Class Model)

Class membership	Probability of being assigned to latent profile				Descriptive	
	Class 1	Class 2	Class 3	Class 4	Range	% <.70
1	**.87**	*.13*	*.00*	*.00*	.43 – 1.00	4 %
2	*.06*	**.91**	*.01*	*.02*	.50 – 1.00	5 %
3	*.00*	*.02*	**.92**	*.06*	.42 – 1.00	1 %
4	*.00*	*.05*	*.03*	**.92**	.47 – 1.00	1 %

Note. High classification quality is determined by high diagonal average posterior probabilities values (as close to 1; in bold) and low off-diagonal values (as close to 0; in italics).[36] *Range indicates the range of posterior probabilities within the specific class. % <.70 indicates the sample proportion with posterior probability of less than .70 with the specific class membership.*

© 2024 Hardi FA et al. *JAMA Network Open.*

eTable 7. Average Posterior Probabilities of the 3-Class and 5-Class Models

**Class membership**	**Probability of latent profile assignment**			**Descriptive**	
	Class 1	Class 2	Class 3	Range	% <.70
1	**.87**	*.13*	*.00*	.43 – 1.00	5 %
2	*.06*	**.90**	*.05*	.46 – 1.00	6 %
3	*.00*	*.09*	**.91**	.50 – 1.00	2 %

**Class membership**	**Probability of latent profile assignment**					**Descriptive**	
	Class 1	Class 2	Class 3	Class 4	Class 5	Range	% < .70
1	**.82**	*.12*	*.06*	*.00*	*.00*	.35 – 1.00	5 %
2	*.10*	**.77**	*.13*	*.00*	*.00*	.36 – 1.00	8 %
3	*.02*	*.05*	**.89**	*.01*	*.02*	.33 – 1.00	5 %
4	*.00*	*.00*	*.03*	**.93**	*.05*	.48 – 1.00	1 %
5	*.00*	*.00*	*.05*	*.04*	**.92**	.49 – 1.00	0.9 %

Note. High classification quality is determined by high diagonal average posterior probabilities values (as close to 1; in bold) and low off-diagonal values (as close to 0; in italics).[36] Range indicates the range of posterior probabilities within the specific class. % <.70 indicates the sample proportion with posterior probability of less than .70 with the specific class membership.

© 2024 Hardi FA et al. *JAMA Network Open.*

eTable 8. Supplementary Latent Profile Analyses (4-Class Model) Leaving One Site Out

	N	AIC	BIC	ABIC	Entropy	Low-adversity *No.* (%)	Medium-adversity *No.* (%)	Maternal Depression *No.* (%)	High-adversity *No.* (%)
All sites	4210	87189.66	87544.99	87367.05	0.82	1230 (29%)	1230 (47%)	550 (13%)	457 (11%)
Site 1 out	3929	81222.94	81574.40	81396.46	0.82	1204 (31%)	1204 (46%)	507 (13%)	401 (10%)
Site 2 out	3928	81504.20	81855.65	81677.71	0.82	1182 (30%)	1182 (46%)	505 (13%)	423 (11%)
Site 3 out	3916	80857.59	81208.87	81030.93	0.82	1167 (30%)	1167 (46%)	522 (13%)	424 (11%)
Site 4 out	3927	80808.92	81160.36	80982.42	0.82	1180 (30%)	1180 (46%)	504 (13%)	430 (11%)
Site 5 out	3936	81203.29	81554.85	81376.91	0.82	1167 (30%)	1167 (46%)	513 (13%)	438 (11%)
Site 6 out	3910	80574.01	80925.20	80747.26	0.82	1160 (30%)	1160 (47%)	495 (13%)	427 (11%)
Site 7 out	3943	81422.73	81774.40	81596.45	0.82	1147 (29%)	1147 (47%)	512 (13%)	422 (11%)
Site 8 out	3914	81318.65	81669.90	81491.96	0.82	1115 (28%)	1115 (47%)	517 (13%)	429 (11%)
Site 9 out	3924	81173.05	81524.44	81346.50	0.82	1169 (30%)	1169 (47%)	517 (13%)	407 (10%)
Site 10 out	3898	80571.40	80922.42	80744.47	0.82	1145 (29%)	1145 (47%)	500 (13%)	415 (11%)
Site 11 out	3898	81485.90	81836.92	81658.97	0.82	1092 (28%)	1092 (47%)	520 (13%)	443 (11%)
Site 12 out	3940	81872.98	82224.60	82046.66	0.82	1179 (30%)	1179 (46%)	531 (13%)	414 (11%)
Site 13 out	4120	85330.99	85685.11	85507.17	0.82	1197 (29%)	1197 (47%)	537 (13%)	443 (11%)
Site 14 out	4124	85470.72	85824.90	85646.96	0.82	1198 (29%)	1198 (47%)	524 (13%)	460 (11%)
Site 15 out	4074	84521.67	84875.16	84697.22	0.82	1199 (29%)	1199 (46%)	542 (13%)	443 (11%)
Site 16 out	4122	85385.23	85739.38	85561.44	0.82	1204 (29%)	1204 (47%)	539 (13%)	442 (11%)
Site 17 out	4121	85481.38	85835.52	85657.57	0.82	1214 (29%)	1214 (47%)	537 (13%)	450 (11%)
Site 18 out	4122	85403.94	85758.09	85580.15	0.82	1150 (28%)	1150 (48%)	528 (13%)	467 (11%)
Site 19 out	4119	85515.74	85869.84	85691.90	0.82	1191 (29%)	1191 (47%)	534 (13%)	456 (11%)
Site 20 out	4125	85416.84	85771.03	85593.08	0.82	1206 (29%)	1206 (47%)	534 (13%)	454 (11%)

Note. AIC indicates Akaike Information Criteria. BIC indicates Bayesian Information Criteria. ABIC indicates adjusted BIC. A list of each site is available on eTable 1 and eTable 2.

© 2024 Hardi FA et al. *JAMA Network Open.*

eTable 9. Descriptives of Each Adversity Latent Profile in the Neuroimaging Subsample (*N*=167)

	**Low-adversity (*n*=38)**	**Medium-adversity (*n*=83)**	**Maternal Depression (*n*=22)**	**High-adversity (*n*=24)**	**Statistical test**
Racial and ethnic identity, No. (%)					
Black (non-Hispanic)	24 (63.2)	66 (79.5)	18 (81.8)	20 (83.3)	$\chi^2(3)$ = 5.17, p = .16
Hispanic	3 (7.9)	8 (9.6)	0 (0)	0 (0)	
Multiracial (non-Hispanic)	1 (2.6)	0 (0)	1 (4.5)	2 (8.3)	
White (non-Hispanic)	8 (21.1)	8 (9.6)	3 (13.6)	1 (4.2)	
Other	2 (5.3)	1 (1.2)	0 (0)	1 (4.2)	
Sex at birth, No. (%)					
Female	26 (68.4)	43 (51.8)	9 (40.9)	13 (54.2)	$\chi^2(3)$ = 4.85, p = .18
Male	12 (31.6)	40 (48.2)	13 (59.1)	11 (45.8)	
Parental marital status, No. (%)					
Married	13 (34.2)	16 (19.3)	6 (27.3)	2 (8.3)	$\chi^2(3)$ = 6.59, p = .09
Unmarried	25 (65.8)	67 (80.7)	16 (72.7)	22 (91.7)	
Poverty ratio *M* (*SD*)	3.33 (3.19)	1.93 (2.03)	1.84 (1.69)	1.04 (1.07)	$F(3,163)$ = 5.97, $p < .001$

Note. Chi-square test for racial and ethnic identity was conducted using two groups (Black vs non). Information about the Other race and ethnicity category is not publicly available.

© 2024 Hardi FA et al. *JAMA Network Open*.

eTable 10. Mean and Standard Deviation of Adversity for Each Profile (N=4210)

**Indicators**	**Low-adversity** ***M (SD)***	**Medium-adversity** ***M (SD)***	**Maternal depression** ***M (SD)***	**High-adversity** ***M (SD)***	***F*** **value**
Emotional abuse	0.69 (0.29)	0.97 (0.37)	1.04 (0.34)	1.11 (0.43)	169.2***
Physical abuse	0.58 (0.38)	0.94 (0.51)	0.98 (0.49)	1.02 (0.58)	124.4***
Neglect	0.09 (0.27)	0.39 (0.41)	0.47 (0.69)	0.58 (1.74)	313***
Maternal depression	0.04 (0.16)	0.27 (0.38)	2.11 (0.59)	1.12 (1.03)	2675***
Intimate partner violence	0.05 (0.16)	0.31 (0.51)	0.33 (0.51)	1.96 (1.83)	666.9***
Parental stress	0.67 (0.30)	0.95 (0.42)	1.08 (0.40)	1.13 (0.48)	242.1***
Residential moves	0.48 (0.40)	0.78 (0.62)	0.91 (0.71)	1.09 (1.05)	127***
Lack of social cohesion	0.55 (0.30)	0.99 (0.41)	0.98 (0.43)	1.20 (0.50)	347.4***
Lack of social control	0.41 (0.34)	0.95 (0.59)	0.83 (0.57)	1.09 (0.73)	236.6***
Neighborhood violence	0.11 (0.23)	0.52 (0.65)	0.51 (0.62)	1.60 (1.57)	341.9***

*Note. Mean and standard deviation above are based on standardized values. ***p < .001.*

© 2024 Hardi FA et al. *JAMA Network Open*.

eTable 11. Mean and Standard Deviation of Adversity in the Neuroimaging Subsample (N=167)

**Indicators**	**Low-adversity** ***M (SD)***	**Medium-adversity** ***M (SD)***	**Maternal depression** ***M (SD)***	**High-adversity** ***M (SD)***	***F* value**
Emotional abuse	0.67 (0.20)	0.93 (0.34)	1.06 (0.29)	1.22 (0.35)	15.6***
Physical abuse	0.60 (0.29)	0.92 (0.43)	0.99 (0.38)	1.17 (0.42)	10.1***
Neglect	0.02 (0.11)	0.26 (0.44)	0.5 (0.55)	1.73 (1.71)	27.55***
Maternal depression	0.02 (0.11)	0.28 (0.37)	1.85 (0.55)	1.40 (0.9)	104***
Intimate partner violence	0.07 (0.22)	0.34 (0.60)	0.55 (0.76)	1.46 (1.62)	13.69***
Parental stress	0.68 (0.28)	0.87 (0.41)	1.04 (0.40)	1.27 (0.35)	13.39***
Residential moves	0.42 (0.36)	0.72 (0.56)	0.89 (0.67)	1.25 (0.97)	9.59***
Lack of social cohesion	0.58 (0.32)	0.95 (0.37)	0.91 (0.36)	1.29 (0.41)	18.21***
Lack of social control	0.40 (0.32)	0.82 (0.57)	0.85 (0.49)	1.29 (0.77)	11.98***
Neighborhood violence	0.14 (0.22)	0.62 (0.62)	0.40 (0.54)	1.70 (0.996)	30.4***

*Note. Mean and standard deviation above are based on standardized values. ***p < .001.*

© 2024 Hardi FA et al. *JAMA Network Open*.

eTable 12. Pairwise Test Comparing Adversity Levels Among Latent Profiles

	**Pairwise contrast**	**Mean difference**	**95% CI Lower bound**	**95% CI Upper bound**	p_{adjust}
Emotional abuse	Med – Low	0.278	0.238	0.318	<.001
	MD – Low	0.348	0.293	0.403	<.001
	High – Low	0.419	0.360	0.479	<.001
	MD – Med	0.070	0.020	0.120	.002
	High – Med	0.141	0.086	0.197	<.001
	High – MD	0.072	0.004	0.139	.03
Physical abuse	Med – Low	0.359	0.305	0.413	<.001
	MD – Low	0.398	0.323	0.473	<.001
	High – Low	0.444	0.363	0.526	<.001
	MD – Med	0.039	-0.030	0.108	.47
	High – Med	0.085	0.009	0.161	.02
	High – MD	0.046	-0.046	0.138	.57
Neglect	Med – Low	0.300	0.213	0.387	<.001
	MD – Low	0.380	0.260	0.500	<.001
	High – Low	1.52	1.391	1.650	<.001
	MD – Med	0.080	-0.030	0.189	.25
	High – Med	1.220	1.100	1.340	<.001
	High – MD	1.141	0.995	1.287	<.001
Maternal depression	Med – Low	0.235	0.189	0.282	<.001
	MD – Low	2.077	2.011	2.142	<.001
	High – Low	1.082	1.012	1.152	<.001
	MD – Med	1.841	1.780	1.903	<.001
	High – Med	0.847	0.781	0.913	<.001
	High – MD	-0.995	-1.075	-0.914	<.001
Intimate partner violence	Med – Low	0.266	0.192	0.340	<.001
	MD – Low	0.283	0.174	0.392	<.001
	High – Low	1.909	1.797	2.022	<.001
	MD – Med	0.017	-0.086	0.120	.97
	High – Med	1.645	1.537	1.751	<.001
	High – MD	1.626	1.493	1.760	<.001
Parental stress	Med – Low	0.284	0.247	0.321	<.001
	MD – Low	0.408	0.356	0.460	<.001
	High – Low	0.462	0.407	0.518	<.001
	MD – Med	0.124	0.076	0.173	<.001
	High – Med	0.178	0.126	0.231	<.001
	High – MD	0.054	-0.010	0.118	.14
Residential moves	Med – Low	0.295	0.235	0.355	<.001
	MD – Low	0.425	0.341	0.510	<.001
	High – Low	0.612	0.522	0.703	<.001
	MD – Med	0.131	0.051	0.210	<.001
	High – Med	0.317	0.232	0.403	<.001
	High – MD	0.187	0.082	0.291	<.001
Lack of social cohesion	Med – Low	0.442	0.400	0.483	<.001
	MD – Low	0.425	0.367	0.483	<.001

© 2024 Hardi FA et al. *JAMA Network Open*.

	High – Low	0.647	0.584	0.710	<.001
	MD – Med	-0.017	-0.037	0.071	.85
	High – Med	0.205	0.146	0.264	<.001
	High – MD	0.222	0.150	0.294	<.001
Lack of social control	Med – Low	0.538	0.481	0.595	<.001
	MD – Low	0.420	0.339	0.500	<.001
	High – Low	0.687	0.600	0.773	<.001
	MD – Med	-0.119	-0.193	-0.044	<.001
	High – Med	0.148	0.067	0.230	<.001
	High – MD	0.267	0.168	0.366	<.001
Neighborhood violence	Med – Low	0.408	0.328	0.489	<.001
	MD – Low	0.397	0.285	0.508	<.001
	High – Low	1.490	1.370	1.610	<.001
	MD – Med	-0.011	-0.091	0.114	.99
	High – Med	1.093	0.971	1.193	<.001
	High – MD	1.082	0.958	1.229	<.001

© 2024 Hardi FA et al. *JAMA Network Open*.

eTable 13. Comparison of Youth Internalizing and Externalizing Among Adversity Profiles

**Internalizing**				
		**95% confidence interval**		
**Contrast**	**Mean Difference**	**Lower bound**	**Upper bound**	**Adjusted *p***
Med – Low	0.188	0.104	0.272	<.001
MD – Low	0.348	0.231	0.466	<.001
High – Low	0.462	0.336	0.589	<.001
Med – MD	-0.160	-0.269	-0.051	.001
High – Med	0.274	0.155	0.393	<.001
High – MD	0.114	-0.030	0.259	.18

**Externalizing**				
		**95% confidence interval**		
**Contrast**	**Mean Difference**	**Lower bound**	**Upper bound**	**Adjusted *p***
Med – Low	0.247	0.175	0.319	<.001
MD – Low	0.370	0.269	0.471	<.001
High – Low	0.496	0.387	0.605	<.001
Med – MD	-0.123	-0.217	-0.029	.004
High – Med	0.249	0.147	0.351	<.001
High – MD	0.126	0.002	0.250	.05

© 2024 Hardi FA et al. *JAMA Network Open.*

eTable 14. Comparison of Youth Internalizing and Externalizing Among Adversity Profiles, Adjusting for Covariates

**Internalizing**					
	**df**	**Sum Sq**	**Mean Sq**	***F* value**	***p* value**
Adversity profiles	3	71	23.65	38.14	< .001
Hispanic	3	71	23.65	38.14	<.001
Multiracial non-Hispanic	1	1.4	1.38	2.23	.14
White non-Hispanic	1	4.3	4.27	6.72	.01
Other	1	0.6	0.57	0.92	.34
Parental marital status	1	6.5	6.46	10.42	.001
Poverty ratio	1	0.3	0.32	0.52	.47
Residuals	3327	2063.4	0.62		

Pairwise test		**95% confidence interval**		
**Contrast**	**Mean Difference**	**Lower bound**	**Upper bound**	**Adjusted *p***
Med – Low	0.19	0.10	0.27	<.001
MD – Low	0.35	0.23	0.47	<.001
High – Low	0.46	0.34	0.59	<.001
Med – MD	-0.16	-0.27	-0.05	.001
High – Med	0.27	0.16	0.39	<.001
High – MD	0.11	-0.03	0.26	.174

Information about the Other race and ethnicity category is not publicly available.

**Externalizing**					
	**df**	**Sum Sq**	**Mean Sq**	***F* value**	***p* value**
Adversity profiles	3	83.2	27.737	61.612	<.001
Hispanic	1	2.10	2.08	4.61	.03
Multiracial non-Hispanic	1	0.70	0.75	1.66	.20
White non-Hispanic	1	2.70	2.67	5.92	.02
Other	1	4.10	4.10	9.11	.003
Parental marital status	1	23.70	23.70	52.65	<.001
Poverty ratio	3	8.80	8.78	19.50	<.001
Residuals	3326	1497.30	0.45		

Pairwise test		**95% confidence interval**		
**Contrast**	**Mean Difference**	**Lower bound**	**Upper bound**	**Adjusted *p***
Med – Low	0.25	0.18	0.32	<.001
MD – Low	0.37	0.27	0.47	<.001
High – Low	0.50	0.39	0.60	<.001
Med – MD	-0.12	-0.22	-0.03	.004
High – Med	0.25	0.15	0.35	<.001

© 2024 Hardi FA et al. *JAMA Network Open*.

High – MD	0.13	0.00	0.25	.042

Information about the Other race and ethnicity category is not publicly available.

eTable 15. Comparison of Functional Connectivity Density Among Adversity Profiles

**Overall Network Density**				
		**95% confidence interval**		
**Contrast**	**Mean Difference**	**Lower bound**	**Upper bound**	**Adjusted *p***
Med – Low	-2.373	-5.608	0.863	.23
MD – Low	-1.450	-5.875	2.975	.83
High – Low	5.868	1.562	10.175	.003
Med – MD	-0.923	-4.884	3.038	.93
High – Med	8.241	4.413	12.069	<.001
High – MD	7.318	2.443	12.194	<.001

**DMN Density**				
		**95% confidence interval**		
**Contrast**	**Mean Difference**	**Lower bound**	**Upper bound**	**Adjusted *p***
Med – Low	-0.005	-0.019	0.008	.72
MD – Low	0.019	0.0002	0.037	.05
High – Low	0.026	0.007	0.044	.002
Med – MD	-0.024	-0.041	-0.008	.001
High – Med	0.031	0.015	0.047	<.001
High – MD	0.007	-0.014	0.027	.83

**SN Density**				
		**95% confidence interval**		
**Contrast**	**Mean Difference**	**Lower bound**	**Upper bound**	**Adjusted *p***
Med – Low	-0.005	-0.018	0.007	.67
MD – Low	-0.010	-0.027	0.008	.50
High – Low	-0.020	-0.037	-0.003	.02
Med – MD	0.004	-0.012	0.020	.91
High – Med	-0.014	-0.030	0.001	.08
High – MD	-0.010	-0.030	0.009	.53

**FPN Density**				
		**95% confidence interval**		
**Contrast**	**Mean Difference**	**Lower bound**	**Upper bound**	**Adjusted *p***
Med – Low	0.016	0.002	0.030	.02
MD – Low	0.005	-0.014	0.024	.90
High – Low	0.052	0.033	0.070	<.001
Med – MD	0.011	-0.006	0.028	.33
High – Med	0.036	0.019	0.052	<.001

© 2024 Hardi FA et al. *JAMA Network Open.*

High – MD	0.047	0.026	0.068	<.001

© 2024 Hardi FA et al. *JAMA Network Open*.

eTable 16. Comparison of Functional Connectivity Density Among Profiles, Adjusting for Covariates

**Overall Network Density**					
	**df**	**Sum Sq**	**Mean Sq**	***F* value**	***p* value**
Adversity profiles	3	1294.0	431.20	12.571 2	<.001
Hispanic	1	76.0	75.50	2.20	.14
Multiracial non-Hispanic	1	0.0	0.30	0.01	.93
White non-Hispanic	1	34.0	33.80	0.99	.32
Other	1	0.0	0.30	0.01	.92
Parental marital status	1	87.0	86.90	2.54	.11
Poverty ratio	1	150.0	150.30	4.38	.04
Age during neuroimaging scan	1	3.0	3.50	0.10	.75
Framewise displacement	1	933.0	933.00	27.202 5	<.001
Residuals	156	5317.0	34.30		

Pairwise test		**95% confidence interval**		
**Contrast**	**Mean Difference**	**Lower bound**	**Upper bound**	**Adjusted *p***
Med – Low	-1.45	-5.52	2.63	.80
MD – Low	-2.37	-5.35	0.61	.17
High – Low	5.87	1.90	9.83	.001
Med – MD	-0.92	-4.57	2.72	.91
High – Med	7.32	2.83	11.81	<.001
High – MD	8.24	4.72	11.77	<.001

Information about the Other race and ethnicity category is not publicly available.

**DMN Density**					
	**df**	**Sum Sq**	**Mean Sq**	***F* value**	***p* value**
Adversity profiles	3	0.024	0.008	11.16	<.001
Hispanic	1	0.000	0.000	0.18	.67
Multiracial non-Hispanic	1	0.001	0.001	1.24	.27
White non-Hispanic	1	0.004	0.004	5.55	.02
Other	1	0.000	0.000	0.05	.82
Parental marital status	1	0.000	0.000	0.23	.63
Poverty ratio	1	0.000	0.000	0.19	.66
Age during neuroimaging scan	1	0.001	0.001	0.82	.37
Framewise displacement	1	0.000	0.000	0.00	.96
Residuals	155	0.111	0.001		

© 2024 Hardi FA et al. *JAMA Network Open.*

Pairwise test Contrast	Mean Difference	95% confidence interval Lower bound	Upper bound	Adjusted *p*
Med – Low	0.019	0.000	0.037	.05
MD – Low	-0.005	-0.019	0.008	.72
High – Low	0.026	0.007	0.044	.002
Med – MD	-0.024	-0.041	-0.008	.001
High – Med	0.007	-0.014	0.027	.83
High – MD	0.031	0.015	0.047	<.001

Information about the Other race and ethnicity category is not publicly available.

**SN Density**					
	**df**	**Sum Sq**	**Mean Sq**	***F* value**	***p* value**
Adversity profiles	3	0.006	0.002	3.22	.02
Hispanic	1	0.002	0.002	3.48	.06
Multiracial non-Hispanic	1	0.001	0.001	1.02	.31
White non-Hispanic	1	0.000	0.000	0.29	.59
Other	1	0.000	0.000	0.34	.56
Parental marital status	1	0.001	0.001	1.91	.17
Poverty ratio	1	0.002	0.002	3.51	.06
Age during neuroimaging scan	1	0.000	0.000	0.08	.78
Framewise displacement	1	0.001	0.001	0.87	.35
Residuals	155	0.098	0.001		

Pairwise test Contrast	Mean Difference	95% confidence interval Lower bound	Upper bound	Adjusted *p*
Med – Low	-0.010	-0.027	0.008	.49
MD – Low	-0.005	-0.018	0.007	.69
High – Low	-0.020	-0.037	-0.003	.02
Med – MD	0.004	-0.012	0.020	.90
High – Med	-0.010	-0.030	0.009	.52
High – MD	-0.014	-0.029	0.001	.07

Information about the Other race and ethnicity category is not publicly available.

**FPN Density**					
	**df**	**Sum Sq**	**Mean Sq**	***F* value**	***p* value**
Adversity profiles	3	0.043	0.014	18.79	<.001
Hispanic	1	0.002	0.002	2.98	.09
Multiracial non-Hispanic	1	0.000	0.000	0.00	.95
White non-Hispanic	1	0.000	0.000	0.55	.46

© 2024 Hardi FA et al. *JAMA Network Open*.

Other	1	0.000	0.000	0.16	.69
Parental marital status	1	0.000	0.000	0.25	.62
Poverty ratio	1	0.000	0.000	0.08	.78
Age during neuroimaging scan	1	0.000	0.000	0.34	.56
Framewise displacement	1	0.002	0.002	2.12	.15
Residuals	155	0.119	0.001		

Pairwise test

**Contrast**	**Mean Difference**	**95% confidence interval**		**Adjusted *p***
		**Lower bound**	**Upper bound**	
Med – Low	0.005	-0.014	0.024	.904
MD – Low	0.016	0.002	0.030	.016
High – Low	0.052	0.033	0.071	<.001
Med – MD	0.011	-0.006	0.028	.330
High – Med	0.047	0.026	0.068	<.001
High – MD	0.036	0.019	0.052	<.001

Information about the Other race and ethnicity category is not publicly available.

© 2024 Hardi FA et al. *JAMA Network Open*.

eTable 17. Comparison of network Connectivity Metrics Estimated Using Neuroimaging Data During Emotional Faces Task vs Resting State Data

**Overall density**	**df**	**SSn**	**SSd**	***F***	**ges**	***p* value**
Between groups	3, 150	2138.24	7609.44	14.05	.136	<.001
Within (scan type)	1, 150	1372.49	6001.06	34.31	.092	<.001
Between:Within	3, 150	93.50	6001.06	0.78	.007	.51

**DMN density**						
	**df**	**SSn**	**SSd**	***F***	**ges**	***p* value**
Between groups	3, 150	0.01	0.12	5.81	.061	<.001
Within (scan type)	1, 150	0.00	0.09	0.03	<.001	.86
Between:Within	3, 150	0.01	0.09	7.52	.063	<.001
Task – Rest pairwise test						
**Profile**	**Estimate**	***SE***	**df**	***t***	**Adjusted *p***	
Low-adversity	0.014	0.006	150	2.329	.02	
Medium-adversity	-0.014	0.004	150	-3.568	.001	
Maternal Depression	0.014	0.008	150	1.891	.06	
High-adversity	-0.012	0.007	150	-1.614	.11	

**SN density**	**df**	**SSn**	**SSd**	**F**	**ges**	***p* value**
Between groups	3, 150	0.01	0.12	3.71	.041	.01
Within (scan type)	1, 150	0.01	0.09	9.71	.026	.002
Between:Within	3, 150	0.01	0.09	5.22	.042	.002
Task – Rest pairwise test						
**Profile**	**Estimate**	***SE***	**df**	***t***	**Adjusted *p***	
Low-adversity	0.008	0.006	150	1.364	.18	
Medium-adversity	-0.005	0.004	150	-1.184	.24	
Maternal Depression	-0.027	0.007	150	-3.696	<.001	
High-adversity	-0.015	0.007	150	-2.068	.04	

**FPN density**	**df**	**SSn**	**SSd**	**F**	**ges**	***p* value**
Between groups	3, 150	0.03	0.11	11.47	.109	<.001
Within (scan type)	1, 150	0.00	0.10	0.26	<.001	.61
Between:Within	3, 150	0.03	0.10	16.56	.133	<.001
Task – Rest pairwise test						
**Profile**	**Estimate**	***SE***	**df**	***t***	**Adjusted *p***	
Low-adversity	-0.034	0.006	150	-5.469	<.001	
Medium-adversity	0.006	0.004	150	1.447	.15	
Maternal Depression	0.003	0.008	150	0.378	.71	
High-adversity	0.032	0.008	150	4.194	<.001	

© 2024 Hardi FA et al. *JAMA Network Open*.

eTable 18. Exploratory Analysis Comparing Youth Internalizing and Externalizing Among Adversity Profiles, Stratified by Sex

**Internalizing (Females)**					
	**df**	**Sum Sq**	**Mean Sq**	***F* value**	***p* value**
Adversity profiles	3	31.200	10.393	15.190	<.001
Residuals	1589	1087.200	0.684		

Pairwise test **Contrast**	**Mean Difference**	**95% confidence interval** **Lower bound**	**Upper bound**	**Adjusted *p***
Med – Low	0.183	0.058	0.309	.001
MD – Low	0.385	0.206	0.565	<.001
High – Low	0.402	0.206	0.597	<.001
Med – MD	-0.202	-0.370	-0.034	.01
High – Med	0.218	0.034	0.403	.01
High – MD	0.016	-0.208	0.241	>.99

**Internalizing (Males)**					
	**df**	**Sum Sq**	**Mean Sq**	***F* value**	***p* value**
Adversity profiles	3	43.200	14.400	25.700	<.001
Residuals	1740	975.000	0.560		

Pairwise test **Contrast**	**Mean Difference**	**95% confidence interval** **Lower bound**	**Upper bound**	**Adjusted *p***
Med – Low	0.195	0.084	0.306	<.001
MD – Low	0.326	0.174	0.479	<.001
High – Low	0.524	0.360	0.687	<.001
Med – MD	-0.131	-0.272	0.010	.08
High – Med	0.329	0.176	0.482	<.001
High – MD	0.198	0.012	0.383	.03

**Externalizing (Females)**					
	**df**	**Sum Sq**	**Mean Sq**	***F* value**	***p* value**
Adversity profiles	3	43.400	14.461	34.630	<.001
Residuals	1589	663.500	0.418		

Pairwise test **Contrast**	**Mean Difference**	**95% confidence interval** **Lower bound**	**Upper bound**	**Adjusted *p***
Med – Low	0.277	0.179	0.375	<.001
MD – Low	0.468	0.328	0.608	<.001
High – Low	0.427	0.274	0.579	<.001

© 2024 Hardi FA et al. *JAMA Network Open*.

Med – MD	-0.191	-0.322	-0.060	.001
High – Med	0.149	0.005	0.293	.04
High – MD	-0.042	-0.217	0.134	.93

**Externalizing (Males)**					
	**df**	**Sum Sq**	**Mean Sq**	***F* value**	***p* value**
Adversity profiles	3	43.100	14.350	29.350	<.001
Residuals	1739	850.100	0.489		

Pairwise test		**95% confidence interval**		
**Contrast**	**Mean Difference**	**Lower bound**	**Upper bound**	**Adjusted *p***
Med – Low	0.216	0.112	0.319	<.001
MD – Low	0.276	0.133	0.418	<.001
High – Low	0.540	0.387	0.693	<.001
Med – MD	-0.060	-0.192	0.072	.65
High – Med	0.324	0.181	0.467	<.001
High – MD	0.264	0.091	0.438	.001

© 2024 Hardi FA et al. *JAMA Network Open*.

eTable 19. Exploratory Analysis Comparing Functional Connectivity Density Among Adversity Profiles, Stratified by Sex

**Overall Network Density (Females)**					
	**df**	**Sum Sq**	**Mean Sq**	***F* value**	***p* value**
Adversity profiles	3	465.600	155.200	5.465	.002
Residuals	87	2470.900	28.400		

Pairwise test Contrast	Mean Difference	95% confidence interval Lower bound	Upper bound	Adjusted *p*
Med – Low	-2.261	-5.729	1.207	.33
MD – Low	-2.863	-8.262	2.536	.51
High – Low	4.154	-0.588	8.896	.11
Med – MD	0.602	-4.515	5.719	.99
High – Med	6.415	1.997	10.833	.001
High – MD	7.017	0.964	13.070	.02

**Overall Network Density (Males)**					
	**df**	**Sum Sq**	**Mean Sq**	***F* value**	***p* value**
Adversity profiles	3	928.000	309.250	5.526	.002
Residuals	72	4029.000	55.960		

Pairwise test Contrast	Mean Difference	95% confidence interval Lower bound	Upper bound	Adjusted *p*
Med – Low	-2.300	-8.776	4.176	.79
MD – Low	-0.212	-8.088	7.664	>.99
High – Low	8.068	-0.144	16.281	.06
Med – MD	-2.088	-8.370	4.193	.82
High – Med	10.368	3.670	17.066	.001
High – MD	8.280	0.220	16.340	.04

**DMN Density (Females)**					
	**df**	**Sum Sq**	**Mean Sq**	***F* value**	***p* value**
Adversity profiles	3	0.014	0.005	5.770	.001
Residuals	87	0.071	0.001		

Pairwise test Contrast	Mean Difference	95% confidence interval Lower bound	Upper bound	Adjusted *p*
Med – Low	-0.009	-0.028	0.009	.57
MD – Low	0.015	-0.014	0.044	.52
High – Low	0.026	0.000	0.051	.05
Med – MD	-0.024	-0.052	0.003	.10

© 2024 Hardi FA et al. *JAMA Network Open.*

High – Med	0.035	0.011	0.058	.001
High – MD	0.010	-0.022	0.043	.84

**DMN Density (Males)**					
	**df**	**Sum Sq**	**Mean Sq**	***F* value**	***p* value**
Adversity profiles	3	0.010	0.003	5.548	.002
Residuals	72	0.045	0.001		

Pairwise test		**95% confidence interval**		
**Contrast**	**Mean Difference**	**Lower bound**	**Upper bound**	**Adjusted *p***
Med – Low	0.000	-0.021	0.022	>.99
MD – Low	0.024	-0.002	0.050	.09
High – Low	0.027	0.000	0.055	.05
Med – MD	-0.024	-0.045	-0.003	.02
High – Med	0.027	0.004	0.049	.01
High – MD	0.003	-0.024	0.030	.99

**SN Density (Females)**					
	**df**	**Sum Sq**	**Mean Sq**	***F* value**	***p* value**
Adversity profiles	3	0.004	0.001	1.692	.18
Residuals	87	0.070	0.001		

**SN Density (Males)**					
	**df**	**Sum Sq**	**Mean Sq**	***F* value**	***p* value**
Adversity profiles	3	0.002	0.001	1.358	.26
Residuals	72	0.035	0.000		

**FPN Density (Females)**					
	**df**	**Sum Sq**	**Mean Sq**	***F* value**	***p* value**
Adversity profiles	3	0.030	0.010	12.800	<.001
Residuals	87	0.067	0.001		

Pairwise test		**95% confidence interval**		
**Contrast**	**Mean Difference**	**Lower bound**	**Upper bound**	**Adjusted *p***
Med – Low	0.016	-0.002	0.034	.11
MD – Low	0.002	-0.026	0.030	>.99
High – Low	0.056	0.032	0.081	<.001
Med – MD	0.014	-0.013	0.040	.53
High – Med	0.041	0.018	0.064	<.001
High – MD	0.054	0.023	0.086	<.001

© 2024 Hardi FA et al. *JAMA Network Open*.

FPN Density (Males)					
	**df**	**Sum Sq**	**Mean Sq**	***F* value**	***p* value**
Adversity profiles	3	0.014	0.005	6.090	.001
Residuals	72	0.056	0.001		

Pairwise test		95% confidence interval		
**Contrast**	**Mean Difference**	**Lower bound**	**Upper bound**	**Adjusted *p***
Med – Low	0.016	-0.008	0.041	.28
MD – Low	0.007	-0.023	0.036	.93
High – Low	0.046	0.016	0.077	.001
Med – MD	0.010	-0.014	0.033	.69
High – Med	0.030	0.005	0.055	.01
High – MD	0.040	0.009	0.070	.01

© 2024 Hardi FA et al. *JAMA Network Open*.

eFigure 1. Exclusionary Criteria for the Neuroimaging Subsample

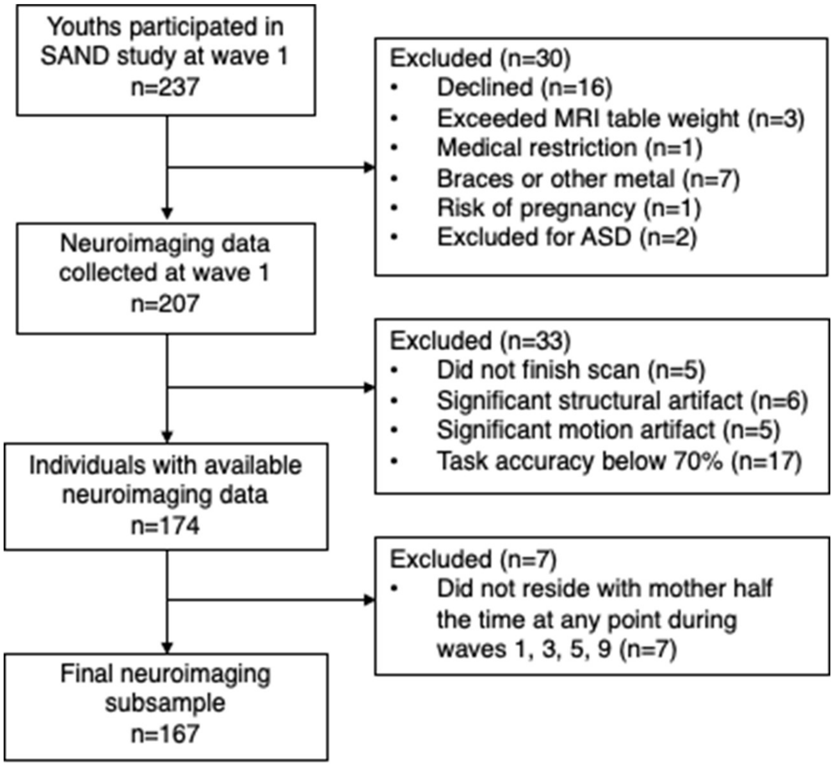

© 2024 Hardi FA et al. *JAMA Network Open*.

eFigure 2. Internalizing Latent Factor Structure and Loadings

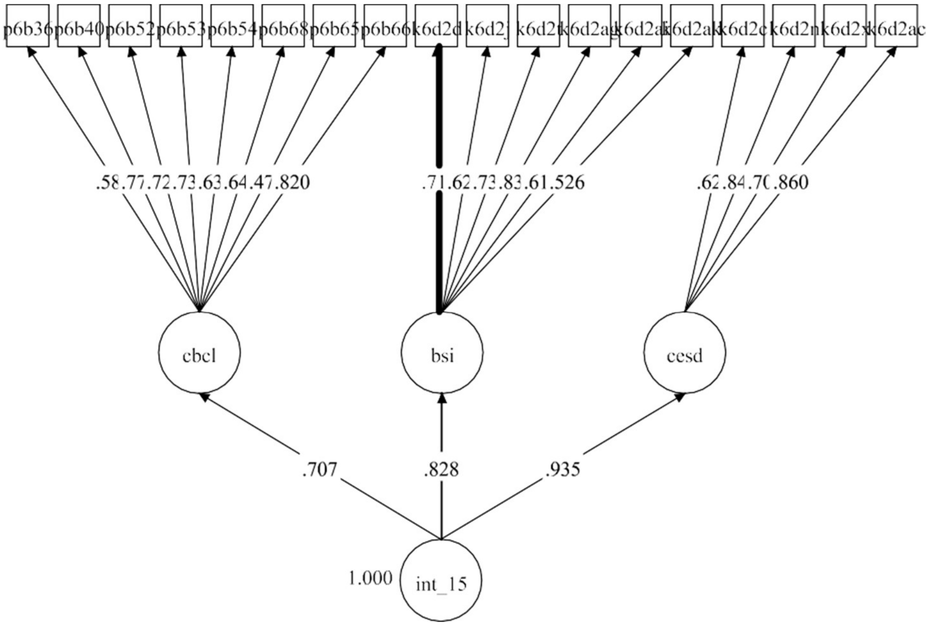

Note. CBCL indicates the Child Behavioral Checklist. BSI indicates the Brief Symptom Inventory 18. CES-D indicates the Center for Epidemiologic Studies Depression Scale. Model fit indices indicate adequate model fit (CFI = .931, TLI = .921, RMSEA = .065, SRMR = .075).

© 2024 Hardi FA et al. *JAMA Network Open*.

eFigure 3. Externalizing Latent Factor Structure and Loadings

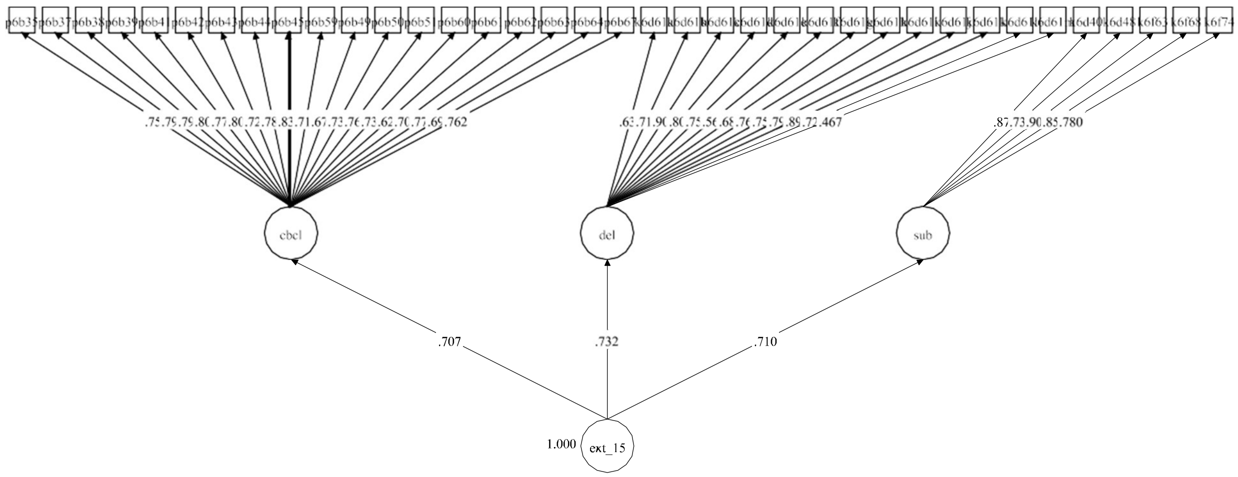

Note. CBCL indicates the Child Behavioral Checklist. DEL indicates the Delinquency scale adopted from the National Longitudinal Study of Adolescent Health (Add Health). SUB indicates youth-reported substance use. Model fit indices indicate excellent model fit (CFI = .955, TLI = .952, RMSEA = .031, SRMR = .092).

© 2024 Hardi FA et al. *JAMA Network Open*.

eFigure 4. Prevalence of Adversity Indicators for the 4-Class Model Within the Neuroimaging Subsample (N=167)

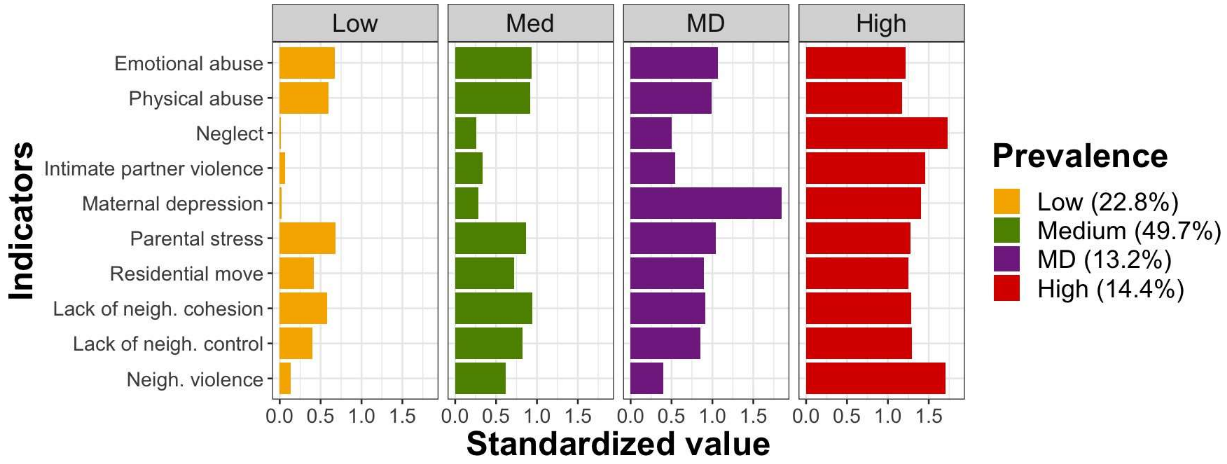

Note. MD denotes Maternal Depression profile

© 2024 Hardi FA et al. *JAMA Network Open*.

eFigure 5. Confirmatory Subgrouping Group Iterative Multiple Model Estimation Network Plots for Each Adversity Profile

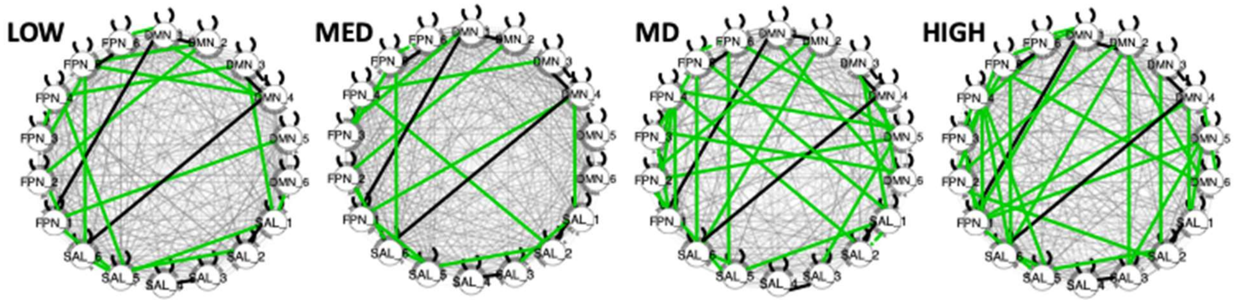

Note. Group-level connections (present for at least 75% of all individuals in the sample) are shown in black. Subgroup-level connections (present for at least 50% of individuals in each latent profile subgroup) are shown in green. Individual-level connections (present for each person) are shown in grey. Specific labels for the ROI represented by individual nodes (e.g., DMN_1) can be found on eTable 3.

© 2024 Hardi FA et al. *JAMA Network Open*.

eFigure 6. Boxplot Showing Network Density Estimated Using Resting-State Functional Neuroimaging Data

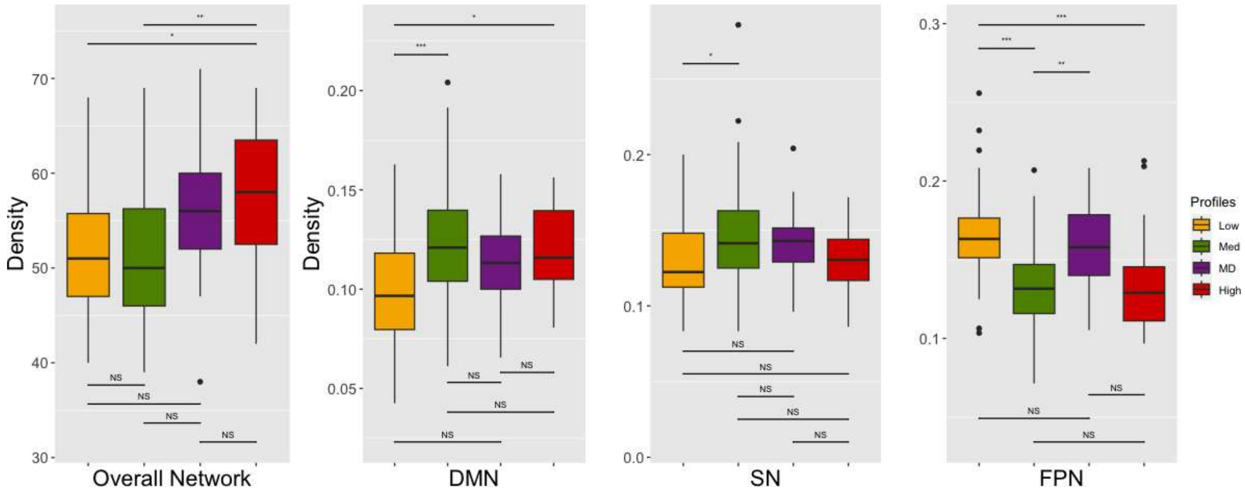

Note. *** $p_{adj} < .001$; ** $p_{adj} < .01$; * $p_{adj} < .05$; NS $p_{adj} > .05$

© 2024 Hardi FA et al. *JAMA Network Open*.

eFigure 7. Youth Mental Health, Stratified by Sex

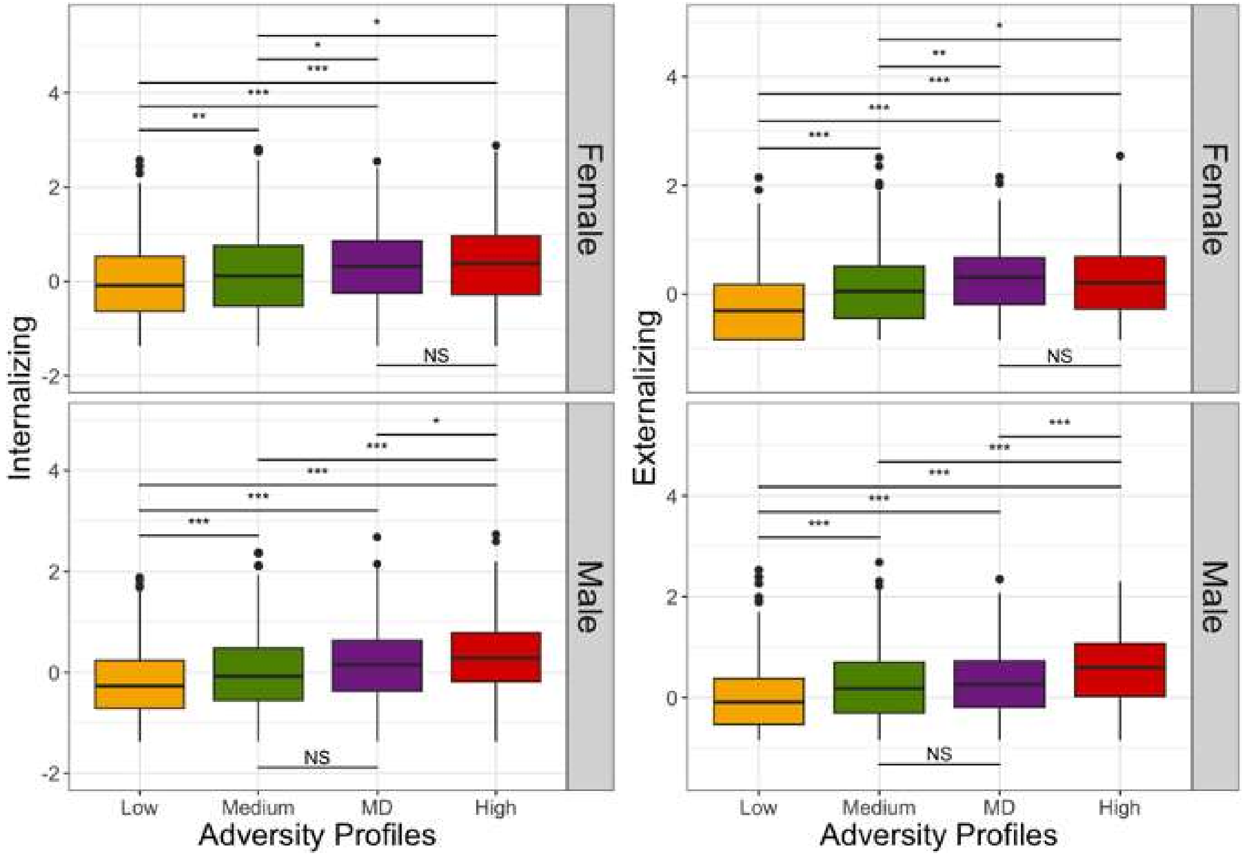

Note. *** $p_{adj} < .001$; ** $p_{adj} < .01$; * $p_{adj} < .05$; NS $p_{adj} > .05$

© 2024 Hardi FA et al. *JAMA Network Open*.

eReferences

1. Goetschius LG, Hein TC, McLanahan SS, et al. Association of Childhood Violence Exposure With Adolescent Neural Network Density. *JAMA Netw Open*. 2020;3(9). doi:10.1001/jamanetworkopen.2020.17850

2. Goetschius LG, Hein TC, Mitchell C, et al. Childhood violence exposure and social deprivation predict adolescent amygdala-orbitofrontal cortex white matter connectivity. *Developmental Cognitive Neuroscience*. 2020;45:100849. doi:10.1016/j.dcn.2020.100849

3. Hardi FA, Goetschius LG, Tillem S, et al. Early childhood household instability, adolescent structural neural network architecture, and young adulthood depression: A 21-year longitudinal study. *Dev Cogn Neurosci*. 2023;61:101253. doi:10.1016/j.dcn.2023.101253

4. Hein TC, Goetschius LG, McLoyd VC, et al. Childhood violence exposure and social deprivation are linked to adolescent threat and reward neural function. *Soc Cogn Affect Neurosci*. 2020;15(11):1252-1259. doi:10.1093/scan/nsaa144

5. Peckins MK, Roberts AG, Hein TC, et al. Violence exposure and social deprivation is associated with cortisol reactivity in urban adolescents. *Psychoneuroendocrinology*. 2020;111:104426. doi:10.1016/j.psyneuen.2019.104426

6. Straus MA, Hamby SL, Finkelhor D, Moore DW, Runyan D. Identification of Child Maltreatment With the Parent-Child Conflict Tactics Scales: Development and Psychometric Data for a National Sample of American Parents. *Child Abuse & Neglect*. 1998;22(4):249-270. doi:10.1016/S0145-2134(97)00174-9

7. Hunt TKA, Berger LM, Slack KS. Adverse Childhood Experiences and Behavioral Problems in Middle Childhood. *Child Abuse Negl*. 2017;67:391-402. doi:10.1016/j.chiabu.2016.11.005

8. Kessler RC, Andrews G, Mroczek D, Ustun B, Wittchen H. The World Health Organization composite international diagnostic interview short-form (CIDI-SF). *International journal of methods in psychiatric research*. 1998;7(4):171-185.

9. Bell CC. DSM-IV: diagnostic and statistical manual of mental disorders. *Jama*. 1994;272(10):828-829.

10. Hofferth S, Davis-Kean PE, Davis J, Finkelstein J. The child development supplement to the Panel Study of Income Dynamics: 1997 user guide. *Ann Arbor: Survey Research Center, Institute for Social Research, University of Michigan*. Published online 1997.

11. Abidin R, Flens JR, Austin WG. *The Parenting Stress Index.* Lawrence Erlbaum Associates Publishers; 2006.

12. Sampson RJ. Collective regulation of adolescent misbehavior: Validation results from eighty Chicago neighborhoods. *Journal of adolescent research*. 1997;12(2):227-244.

© 2024 Hardi FA et al. *JAMA Network Open*.

13. Sampson RJ, Raudenbush SW, Earls F. Neighborhoods and Violent Crime: A Multilevel Study of Collective Efficacy. *Science*. 1997;277(5328):918-924. doi:10.1126/science.277.5328.918

14. Zhang S, Anderson SG. Low-income single mothers' community violence exposure and aggressive parenting practices. *Children and Youth Services Review*. 2010;32(6):889-895.

15. Achenbach TM. Manual for ASEBA school-age forms & profiles. *University of Vermont, Research Center for Children, Youth & Families*. Published online 2001.

16. Derogatis LR, Kathryn L. The SCL-90-R and Brief Symptom Inventory (BSI) in primary care. In: *Handbook of Psychological Assessment in Primary Care Settings*. Routledge; 2000:310-347.

17. Radloff LS. The CES-D scale: A self-report depression scale for research in the general population. *Applied psychological measurement*. 1977;1(3):385-401.

18. Muthén B, Muthén L. Mplus. In: *Handbook of Item Response Theory*. Chapman and Hall/CRC; 2017:507-518.

19. Hu L tze, Bentler PM. Cutoff criteria for fit indexes in covariance structure analysis: Conventional criteria versus new alternatives. *Structural Equation Modeling*. 1999;6(1):1-55. doi:10.1080/10705519909540118

20. Harris KM. The add health study: Design and accomplishments. *Chapel Hill: Carolina Population Center, University of North Carolina at Chapel Hill*. 2013;1:1-22.

21. Reichman NE, Teitler JO, Garfinkel I, McLanahan S. Fragile Families: Sample and Design. *Children and Youth Services Review*. 2001;23. doi:10.1016/s0190-7409(01)00141-4

22. Power JD, Barnes KA, Snyder AZ, Schlaggar BL, Petersen SE. Spurious but systematic correlations in functional connectivity MRI networks arise from subject motion. *Neuroimage*. 2012;59(3):2142-2154. doi:10.1016/j.neuroimage.2011.10.018

23. Liu C, Rubin DonaldB. Ellipsoidally symmetric extensions of the general location model for mixed categorical and continuous data. *Biometrika*. 1998;85(3):673-688. doi:10.1093/biomet/85.3.673

24. Proust-Lima C, Philipps V, Liquet B. Estimation of Extended Mixed Models Using Latent Classes and Latent Processes: The R Package lcmm. *Journal of Statistical Software*. 2017;78:1-56. doi:10.18637/jss.v078.i02

25. McNeish D, Harring JR, Bauer DJ. Nonconvergence, covariance constraints, and class enumeration in growth mixture models. *Psychological Methods*. Published online 2022.

26. Barnard J, McCulloch R, Meng XL. Modeling covariance matrices in terms of standard deviations and correlations, with application to shrinkage. *Statistica Sinica*. Published online 2000:1281-1311.

© 2024 Hardi FA et al. *JAMA Network Open*.

27. Nylund KL, Asparouhov T, Muthén BO. Deciding on the number of classes in latent class analysis and growth mixture modeling: A Monte Carlo simulation study. *Structural Equation Modeling*. 2008;15(1):182-182. doi:10.1080/10705510701793320

28. Faubert BEW Natasha K Bowen, Sarah J. Latent Class Analysis: A Guide to Best Practice - Bridget E. Weller, Natasha K. Bowen, Sarah J. Faubert, 2020. *Journal of Black Psychology*. Published online June 5, 2020. Accessed March 20, 2024. https://journals.sagepub.com/doi/full/10.1177/0095798420930932

29. Sinha P, Calfee CS, Delucchi KL. Practitioner's Guide to Latent Class Analysis: Methodological Considerations and Common Pitfalls. *Critical care medicine*. 2021;49(1):e63. doi:10.1097/CCM.0000000000004710

30. Akaike H. Factor analysis and AIC. *Psychometrika*. 1987;52:317-332.

31. Schwarz G. Estimating the dimension of a model. *The annals of statistics*. Published online 1978:461-464.

32. Sclove SL. Application of model-selection criteria to some problems in multivariate analysis. *Psychometrika*. 1987;52:333-343.

33. Lo Y, Mendell NR, Rubin DB. Testing the number of components in a normal mixture. *Biometrika*. 2001;88(3):767-778. doi:10.1093/biomet/88.3.767

34. Masyn KE. Latent Class Analysis and Finite Mixture Modeling. In: Little TD, ed. *The Oxford Handbook of Quantitative Methods in Psychology: Vol. 2: Statistical Analysis*. Oxford University Press; 2013:0. doi:10.1093/oxfordhb/9780199934898.013.0025

35. Celeux G, Soromenho G. An entropy criterion for assessing the number of clusters in a mixture model. *Journal of Classification*. 1996;13(2):195-212. doi:10.1007/BF01246098

36. Muthén B, Muthén LK. Integrating Person-Centered and Variable-Centered Analyses: Growth Mixture Modeling With Latent Trajectory Classes. *Alcohol: Clinical and Experimental Research*. 2000;24(6):882-891. doi:10.1111/j.1530-0277.2000.tb02070.x

37. Nagin DS. Group-Based Modeling of Development. In: *Group-Based Modeling of Development*. Harvard University Press; 2009. doi:10.4159/9780674041318

38. Glover GH, Law CS. Spiral-in/out BOLD fMRI for increased SNR and reduced susceptibility artifacts. *Magnetic Resonance in Medicine*. 2001;46(3):515-522. doi:10.1002/mrm.1222

39. Hardi FA, Goetschius LG, McLoyd V, et al. Adolescent functional network connectivity prospectively predicts adult anxiety symptoms related to perceived COVID-19 economic adversity. *Journal of Child Psychology and Psychiatry*. 2023;64(6):918-929. doi:10.1111/jcpp.13749

© 2024 Hardi FA et al. *JAMA Network Open*.

40. Tottenham N, Tanaka JW, Leon AC, et al. The NimStim set of facial expressions: judgments from untrained research participants. *Psychiatry Res*. 2009;168(3):242-249. doi:10.1016/j.psychres.2008.05.006

41. Jenkinson M, Beckmann CF, Behrens TEJ, Woolrich MW, Smith SM. FSL. *Neuroimage*. 2012;62(2):782-790. doi:10.1016/j.neuroimage.2011.09.015

42. Woolrich MW, Ripley BD, Brady M, Smith SM. Temporal autocorrelation in univariate linear modeling of FMRI data. *Neuroimage*. 2001;14(6):1370-1386. doi:10.1006/nimg.2001.0931

43. Adriene M. Beltz, Hailey L. Dotterer, Leigh G. Goetschius. GIMME Preprocessing: Initial Release. Published online May 9, 2019. doi:10.5281/zenodo.2692522

44. Raichle ME. The Brain's Default Mode Network. *Annual Review of Neuroscience*. 2015;38(1):433-447. doi:10.1146/annurev-neuro-071013-014030

45. Raichle ME, MacLeod AM, Snyder AZ, Powers WJ, Gusnard DA, Shulman GL. A default mode of brain function. *PNAS*. 2001;98(2):676-682. doi:10.1073/pnas.98.2.676

46. Seeley WW. The Salience Network: A Neural System for Perceiving and Responding to Homeostatic Demands. *J Neurosci*. 2019;39(50):9878-9882. doi:10.1523/JNEUROSCI.1138-17.2019

47. Menon V. Large-scale brain networks and psychopathology: a unifying triple network model. *Trends in Cognitive Sciences*. 2011;15(10):483-506. doi:10.1016/j.tics.2011.08.003

48. Zanto TP, Gazzaley A. Fronto-parietal network: flexible hub of cognitive control. *Trends in Cognitive Sciences*. 2013;17(12):602-603. doi:10.1016/j.tics.2013.10.001

49. Yarkoni T, Poldrack RA, Nichols TE, Van Essen DC, Wager TD. Large-scale automated synthesis of human functional neuroimaging data. *Nat Methods*. 2011;8(8):665-670. doi:10.1038/nmeth.1635

50. Henry TR, Feczko E, Cordova M, et al. Comparing directed functional connectivity between groups with confirmatory subgrouping GIMME. *NeuroImage*. 2019;188:642-653. doi:10.1016/j.neuroimage.2018.12.040

51. Gates KM, Molenaar PCM. Group search algorithm recovers effective connectivity maps for individuals in homogeneous and heterogeneous samples. *NeuroImage*. 2012;63(1):310-319. doi:10.1016/j.neuroimage.2012.06.026

52. Beltz AM, Gates KM. Network Mapping with GIMME. *Multivariate Behavioral Research*. 2017;52(6):789-804. doi:10.1080/00273171.2017.1373014

53. Gates KM, Molenaar PCM, Hillary FG, Ram N, Rovine MJ. Automatic search for fMRI connectivity mapping: an alternative to Granger causality testing using formal equivalences

among SEM path modeling, VAR, and unified SEM. *Neuroimage*. 2010;50(3):1118-1125. doi:10.1016/j.neuroimage.2009.12.117

© 2024 Hardi FA et al. *JAMA Network Open*.
