# Supplementary material for: Latent Profiles of Childhood Adversity, Adolescent Mental Health, and Neural Network Connectivity
Source: JAMA Netw Open. 2024 Aug 28;7(8):e2430711. doi: 10.1001/jamanetworkopen.2024.30711 (PMC11358864; doi:10.1001/jamanetworkopen.2024.30711)
Supplement: Supplement 2. — Data Sharing Statement [file jamanetwopen-e2430711-s002.pdf]

Data Sharing Statement

Hardi. Latent Profiles of Childhood Adversity, Adolescent Mental Health, and Neural Network Connectivity. *JAMA Netw Open*. Published August 28, 2024.
doi:10.1001/jamanetworkopen.2024.30711

Data
Data available: Yes
Data types: Deidentified participant data
How to access data: https://nda-nih-gov.proxy.lib.umich.edu/edit_collection.html?id=2106
When available: With publication

Supporting Documents
Document types: Statistical/analytic code, Informed consent form
How to access documents: https://nda-nih-gov.proxy.lib.umich.edu/edit_collection.html?id=2106
When available: With publication

Additional Information
Who can access the data: Anyone requesting the data
Types of analyses: For a specified purpose
Mechanisms of data availability: With a signed data access agreement
